# Ground-Level NO_2_ Surveillance from Space Across China for High Resolution Using Interpretable Spatiotemporally Weighted Artificial Intelligence

**DOI:** 10.1021/acs.est.2c03834

**Published:** 2022-06-29

**Authors:** Jing Wei, Song Liu, Zhanqing Li, Cheng Liu, Kai Qin, Xiong Liu, Rachel T. Pinker, Russell R. Dickerson, Jintai Lin, K. F. Boersma, Lin Sun, Runze Li, Wenhao Xue, Yuanzheng Cui, Chengxin Zhang, Jun Wang

**Affiliations:** † Department of Chemical and Biochemical Engineering, Iowa Technology Institute, Center for Global and Regional Environmental Research, University of Iowa, Iowa City, Iowa 52242, United States; ‡ Department of Atmospheric and Oceanic Science, Earth System Science Interdisciplinary Center, University of Maryland, College Park, Maryland 20742, United States; § School of Environmental Science and Engineering, Southern University of Science and Technology, Shenzhen 518055, China; ∥ Department of Precision Machinery and Precision Instrumentation, University of Science and Technology of China, Hefei 230026, China; ⊥ School of Environment and Geoinformatics, China University of Mining and Technology, Xuzhou 221116, China; # Atomic and Molecular Physics Division, Center for Astrophysics | Harvard and Smithsonian, Cambridge, Massachusetts 02138, United States; ∇ Laboratory for Climate and Ocean-Atmosphere Studies, Department of Atmospheric and Oceanic Sciences, School of Physics, Peking University, Beijing 100871, China; ○ Satellite Observations Department, Royal Netherlands Meteorological Institute, De Bilt 3731GA, the Netherlands; ◆ Meteorology and Air Quality Group, Wageningen University, Wageningen 6708PB, the Netherlands; ¶ College of Geodesy and Geomatics, Shandong University of Science and Technology, Qingdao 266590, China; †† Department of Civil and Environmental Engineering, University of California, Irvine, California 92697, United States; ‡‡ School of Economics, Qingdao University, Qingdao 266071, China; §§ College of Hydrology and Water Resources, Hohai University, Nanjing 210098, China

**Keywords:** surface NO_2_, air pollution, big data, artificial intelligence, COVID-19

## Abstract

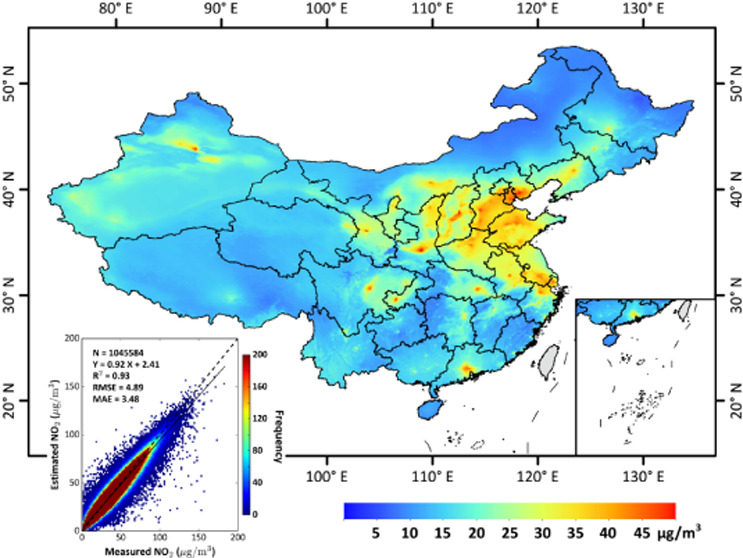

Nitrogen dioxide (NO_2_) at the ground level poses a serious threat to environmental quality and public health. This study developed a novel, artificial intelligence approach by integrating spatiotemporally weighted information into the missing extra-trees and deep forest models to first fill the satellite data gaps and increase data availability by 49% and then derive daily 1 km surface NO_2_ concentrations over mainland China with full spatial coverage (100%) for the period 2019–2020 by combining surface NO_2_ measurements, satellite tropospheric NO_2_ columns derived from TROPOMI and OMI, atmospheric reanalysis, and model simulations. Our daily surface NO_2_ estimates have an average out-of-sample (out-of-city) cross-validation coefficient of determination of 0.93 (0.71) and root-mean-square error of 4.89 (9.95) μg/m^3^. The daily seamless high-resolution and high-quality dataset “ChinaHighNO_2_” allows us to examine spatial patterns at fine scales such as the urban–rural contrast. We observed systematic large differences between urban and rural areas (28% on average) in surface NO_2_, especially in provincial capitals. Strong holiday effects were found, with average declines of 22 and 14% during the Spring Festival and the National Day in China, respectively. Unlike North America and Europe, there is little difference between weekdays and weekends (within ±1 μg/m^3^). During the COVID-19 pandemic, surface NO_2_ concentrations decreased considerably and then gradually returned to normal levels around the 72nd day after the Lunar New Year in China, which is about 3 weeks longer than the tropospheric NO_2_ column, implying that the former can better represent the changes in NO*_x_* emissions.

## Abstract 2

Daily seamless 1 km resolution high-quality surface NO_2_ dataset across mainland China is generated from big data using artificial intelligence.

## 1 Introduction

Nitrogen dioxide (NO_2_) is one of the most important trace gases in the atmosphere, greatly impacting the ecological environment and air quality.^1−4^ It is a major pollutant near the ground and can be inhaled, posing a health threat.^5,6^ Nitrogen oxides (NO*_x_* = NO_2_ + NO) are PM_2.5_ precursors in haze and lead to the formation of surface ozone.^7,8^ NO*_x_* comes from diverse and complex sources including fossil-fuel-fired power plants, automobile exhaust, industrial activities, biofuel, and resident cooking; the major natural sources are wildfires, soil, and lightning.^9^

Surface NO_2_ levels are an important measure of air quality/pollution. Because NO*_x_* has a short atmospheric lifetime, it is challenging to accurately quantify surface NO_2_ concentrations with good spatial coverage based on sparse ground-based monitoring stations, especially in developing countries with dense populations, like China. Satellite remote sensing allows monitoring of global NO_2_ distributions and variations such as with the global ozone monitoring experiment (GOME) instrument^10^ and the ozone monitoring instrument (OMI).^11,12^ However, satellites can provide only the total or tropospheric NO_2_ columns. Attempts have been made to convert satellite tropospheric NO_2_ retrievals to ground-level concentrations using different chemical transport^13^ and statistical models. Qin et al. adopted the geographically and temporally weighted regression (GTWR) and extremely randomized trees (ERT) models to derive daily surface NO_2_ from OMI tropospheric NO_2_ products and meteorological data in central–eastern China.^14,15^ The daily ambient NO_2_ exposure was also estimated in China from OMI tropospheric NO_2_ products and environmental data by combining the universal kriging or the K-means approaches with land-use regression (LUR)^16^ and different machine-learning models (e.g., random forest or RF).^17−19^ Li and Wu applied the deep learning of full residual deep networks (FSDN) to obtain the outdoor NO_2_ concentrations in China by incorporating OMI tropospheric NO_2_ and other covariates.^20^

These surface NO_2_ estimates were generated from OMI on the Aura satellite with spatial resolutions, 13 km × 24 km at nadir and up to ∼26 km × 160 km at the edge of the swath, which cannot resolve high variations of surface NO_2_ at medium or small (urban) scales.^21,22^ The Tropospheric Monitoring Instrument (TROPOMI) is onboard the copernicus sentinel-5 precursor (S5P) satellite, launched on 13 October 2017, and provides higher-spatial-resolution tropospheric NO_2_ retrievals (e.g., 3.5 km × 5.5 km after August 2019). So far, only a handful of studies have employed the TROPOMI data to estimate near-surface high-resolution NO_2_ concentration-adopted statistical regression (e.g., GTWR) and machine-learning [e.g., light gradient boosting machine (LightGBM) and extreme gradient boosting (XGBoost)] models.^23−25^

Most previous studies have simply directly applied traditional methods that ignored the spatiotemporal heterogeneity of air pollution, especially machine learning that works via the single-pixel-based processing mode.^26^ This leads to an inhomogeneous distribution of air pollution with the nonsmooth transition or the discontinuity of air pollution mass in the space (and possibly yielding “salt and pepper” white noises in the data). In addition, the weaker signals of trace gases bring greater difficulties in both satellite gap filling and ground-level air pollutant estimation. It is thus necessary to consider additional factors such as model simulations and emission inventories to develop more accurate methods to improve accuracy. Here, a novel framework integrating machine and deep-learning models by involving spatiotemporally weighted characteristics of air pollution is developed to fill missing values in satellite tropospheric NO_2_ products and derive daily seamless 1 km resolution ground-level NO_2_ concentrations from rich big data across mainland China. Last, the application and fidelity of the dataset are demonstrated by analyzing the spatial distributions of surface NO_2_ across China and their variations during statutory holidays and the COVID-19 pandemic.

## 2 Materials and Methods

### 2.1 Big Data

Data used in our study include ground-based in situ observations, satellite remote sensing products, atmospheric reanalysis, and model simulation. Hourly ground-level NO_2_ concentrations (unit: μg/m^3^) measured at ∼1630 monitoring stations across mainland China from 1 January 2019 to 31 December 2020 were collected from the Chinese Ministry of Environment and Ecology. Daily means were calculated from hourly observations that had undergone additional quality control measures,^27^ i.e., filtering out invalid values and outliers caused by suspected instrument malfunction (details provided in Supporting Text 1).

Daily TROPOMI tropospheric NO_2_ products (unit: 1e15 mol/cm^2^) at a high spatial resolution of 1 km in China generated using a new algorithm and downscaled following the area-weighted method were employed.^28^ This approach has significantly reduced the uncertainty compared to official products, especially in highly polluted areas that had been severely underestimated.^29−31^ Here, the recommended good-quality (quality assurance value > 0.75) tropospheric vertical column NO_2_ retrievals were selected. In addition, new daily OMI tropospheric NO_2_ products (0.25 × 0.25°) reconstructed in mainland China^32^ were employed. NO_2_ simulations (0.75 × 0.75°) every 1 h modeled at tropospheric and ground levels were calculated from the CAMS global reanalysis, and monthly anthropogenic NO*_x_* emissions (0.1 × 0.1°) were obtained from the CAMS global emission inventory.^33^

The ERA5 hourly atmospheric reanalysis product contains eight meteorological fields, i.e., boundary layer height (BLH), 2 m temperature (TEM), evaporation (ET), precipitation (PRE), relative humidity (RH), 10 m u-component of wind (WU), 10 m v-component of wind (WV), and surface pressure (SP), collected from the ERA5 hourly atmospheric reanalysis products.^34,35^ Population-related data include the 1 km landScan population distribution (POD) and 1 km visible infrared imaging radiometer suite nighttime light (NTL) products. Land-surface-related data include the moderate resolution imaging spectroradiometer (MODIS) land-use type (500 m) and normalized difference vegetation index (NDVI, 1 km) products and the shuttle radar topography mission (SRTM) digital elevation model (DEM, 90 m) products. Table S1 summarizes the detailed information on big data used in our study, and all of the auxiliary data were regridded to a uniform spatial resolution of 0.01 × 0.01° (≈1 km × 1 km).

### 2.2 Methodology

We combine machine- and deep-learning models that consider the spatiotemporal heterogeneity of air pollution. The model framework includes a spatiotemporally weighted missing extra-trees (SWMET) model for filling the satellite tropospheric NO_2_ gaps and a spatiotemporally weighted deep forest (SWDF) model to estimate surface NO_2_.

#### 2.2.1 Tropospheric NO_2_ Gap Filling

Ubiquitous clouds in optical remote sensing images prevent tropospheric below-cloud NO_2_ information from being detected due to shielding. An efficient machine-learning model, named missing extra-trees (MET),^36^ was adopted to fill satellite data gaps. Differing from traditional methods (e.g., inverse distance weight or kriging), MET is a nonparametric spatial interpolation method that works like the missing forest^37^ but with stronger randomizations, which can impute a dataset with missing values in multiple variables using an iterative way. It belongs to tree-based ensemble learning, which has a strong antinoise capability and is insensitive to multivariate collinearity. Here, spatiotemporal autocorrelations in satellite tropospheric NO_2_ retrievals were considered in the MET model, leading to a new spatiotemporally weighted extra-trees (SWMET) model. This model was used to impute missing values in OMI and TROPOMI tropospheric NO_2_ retrievals in sequence through two iterations, respectively, together with other spatiotemporally continuous auxiliary variables with potential influence (details provided in Supporting Text 2).

#### 2.2.2 Ground-Level NO_2_ Estimation

Considering the more complex and weaker relationships with tropospheric signals, deep learning (more flexible with a higher capability) was employed to estimate ground-level NO_2_ concentrations. Deep forest (DF), developed by Zhou and Feng in 2017^38^ and recently updated in 2021, was adopted. Based on the idea of deep neural networks that stack neural networks, DF stacks multilayer RFs and completely random tree forests in a cascade to obtain better feature representation and learning performance. DF can handle data of different scales without setting super parameters and has more competitive performance and a better physical interpretation than other “black box” deep-learning models. We also introduce a new SWDF model that incorporates the spatiotemporally weighted information into the DF model to construct a robust tropospheric-surface NO_2_ conversion model involving all potential influencing factors (details provided in Supporting Text 3). Here, the model was trained and built each year separately for ground-level NO_2_ estimations in China.

#### 2.2.3 Spatiotemporal Weight Information

Temporally, air pollution can have strong seasonal cycles, and concentrations vary on the daily as well as synoptic scales, which may be similar on adjacent days, but differences increase as the time interval increases. In addition, such short-lived species also vary significantly spatially, where the farther away the two points are, the more different the polluted level is. Such differences in time and space are often not equal. Therefore, in this study, different from our previous studies that only considered the equal effects,^39,40^ spatiotemporally weighted information at different spatial points was updated and incorporated in the original artificial intelligence models to better distinguish spatiotemporal differences in air pollutants and improve their estimates.

The spatial term (*P*_s_) is represented by the latitude (Lat) and longitude (Lon) information of one point, and the square root of inverse Haversine great-circle distances^41^ from the point to the corners in eight directions, i.e., top-left (*D*_p1_), top-middle (*D*_p2_), top-right (*D*_p3_), right-middle (*D*_p4_), bottom-right (*D*_p5_), bottom-middle (*D*_p6_), bottom-left (*D*_p7_), and left-middle (*D*_p8_), and the center (*D*_p9_) of the study area (eq 1). The temporal term (*P*_t_) is represented by the day of the year (DOY), and the inverse distances  from 1 day to the middle day of four seasons, i.e., spring equinox (21 March, *D*_t1_), summer solstice (21 June, *D*_t2_), autumn equinox (22 September, *D*_t3_), and winter solstice (22 December, *D*_t4_) (eq 2).
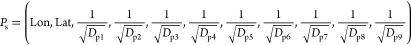
1
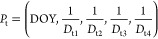
2

#### 2.2.4 Validation Method

The widely used out-of-sample 10-fold cross-validation (10 CV) method, where data samples are randomly divided into 10 folds, saving one fold for testing, was selected to evaluate the overall accuracy of surface NO_2_ estimates.^42^ In addition, considering the use of information from neighboring stations, a new independent out-of-city 10 CV approach, where cities are randomly divided into 10 folds, saving one fold for testing, was employed to assess the model’s predictive ability in space, i.e., surface NO_2_ predictions at locations where ground-based measurements are not available.

## 3 Results and Discussion

### 3.1 Model Performance

#### 3.1.1 Tropospheric and Surface NO_2_ Results

**Figure 1:**
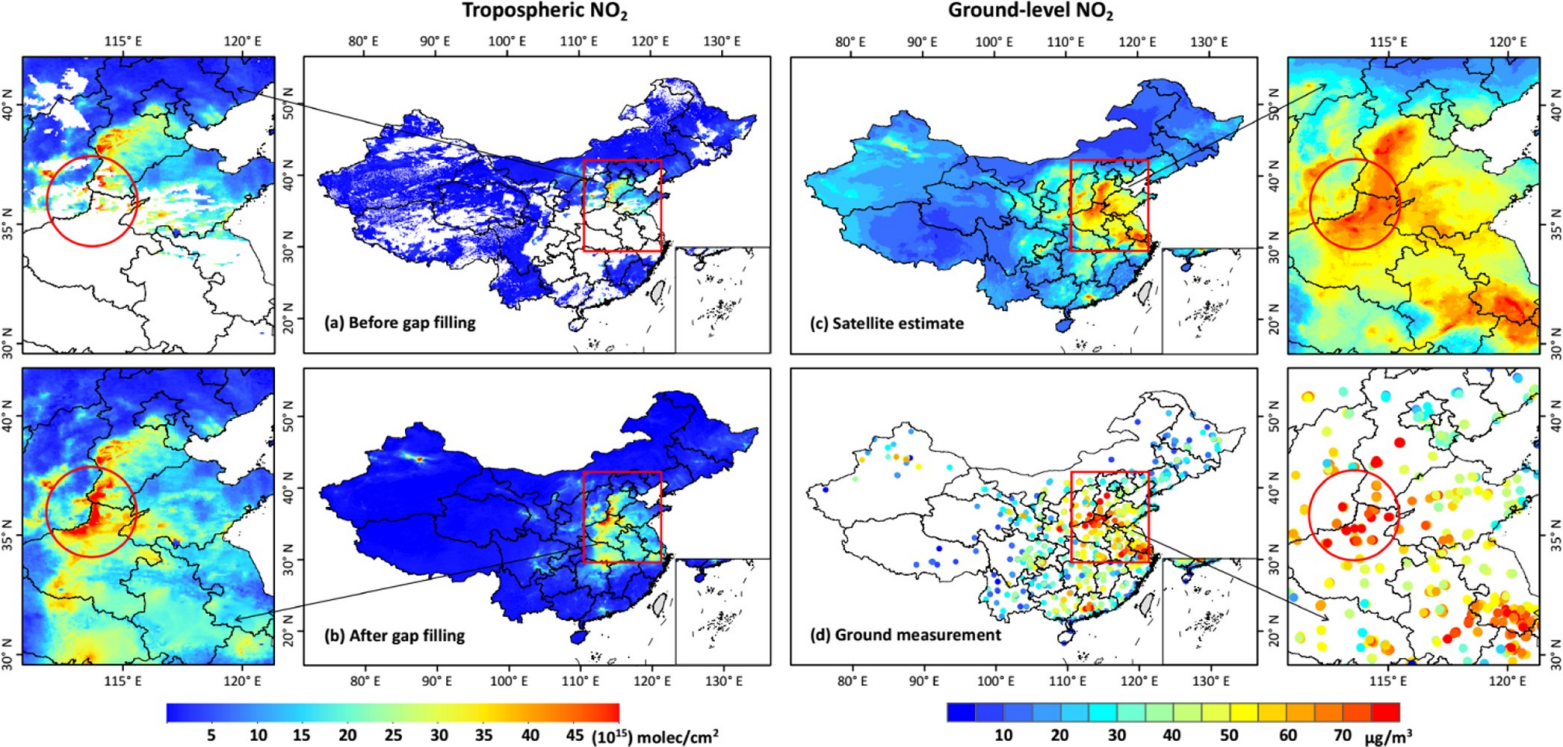
National and regional (zoomed-in subplots) spatial distributions of (a) original and (b) gap-filled TROPOMI tropospheric NO_2_ columns (mol/cm^2^, bottom-left legend), and (c) our model-derived and (d) ground-measured surface NO_2_ concentrations (μg/m^3^, bottom-right legend) on 28 January 2019 in China. Red circles in the subplots outline areas of heavy pollution.

The OMI tropospheric NO_2_ product has nearly complete spatial coverage (average = 87%) after preliminary data fusion by integrating global ozone monitoring experiment-2B (GOME-2B) NO_2_ information using a reconstructed framework (Figure S1a).^32^ In contrast, TROPOMI tropospheric NO_2_ products have a large number of missing retrievals with an average spatial coverage of only about 51%, especially in the pluvial areas of southern China (Figure S1b). Applying the SWMET model, daily 1 km tropospheric NO_2_ maps with a spatial coverage increasing to 100% covering mainland China were generated. In general, tropospheric NO_2_ information in missing areas can be well reconstructed (Figure 1a,b) even under highly polluted conditions (areas outlined by red circles).

Figure 1c shows a typical example of the spatial distribution of our model-derived ground-level NO_2_ concentrations on an individual day, i.e., 28 January 2019. By filling satellite gaps, our ChinaHighNO_2_ dataset can provide surface NO_2_ information at any location throughout the country. Compared with ground measurements, our gap-filled dataset can also capture the spatial distributions of surface NO_2_ in lightly polluted areas, e.g., northeast and southwest China (Figure 1d). More importantly, our predictions are highly consistent with observations both in spatial patterns and magnitudes over severely NO_2_-polluted areas in eastern China, especially the North China Plain, where a large number of missing values existed in satellite tropospheric data (Figure 1a). This further illustrates the superior performance of our gap-filling method under polluted conditions.

#### 3.1.2 Validation of NO_2_ Estimates and Predictions

Gap-filled tropospheric NO_2_ data compare well with multiaxis differential optical absorption spectroscopy (MAX-DOAS) measurements (only a slightly decreased correlation of 0.72 from 0.85 compared to nongap-filled retrievals) at the individual Xuzhou site (34.22° N, 117.14° E). However, limited by the available number of ground monitors, the same independent cross-validation method adopted in previous studies^20,24^ was selected to further validate gap-filling data. The results illustrate that our tropospheric NO_2_ predictions are reliable, with the average coefficient of determination (*R*^2^) values ranging from 0.89 to 0.96 and root-mean-square error (RMSE) values ranging from 0.46 × 10^15^ to 1.51 × 10^15^ mol/cm^2^.

**Figure 2:**
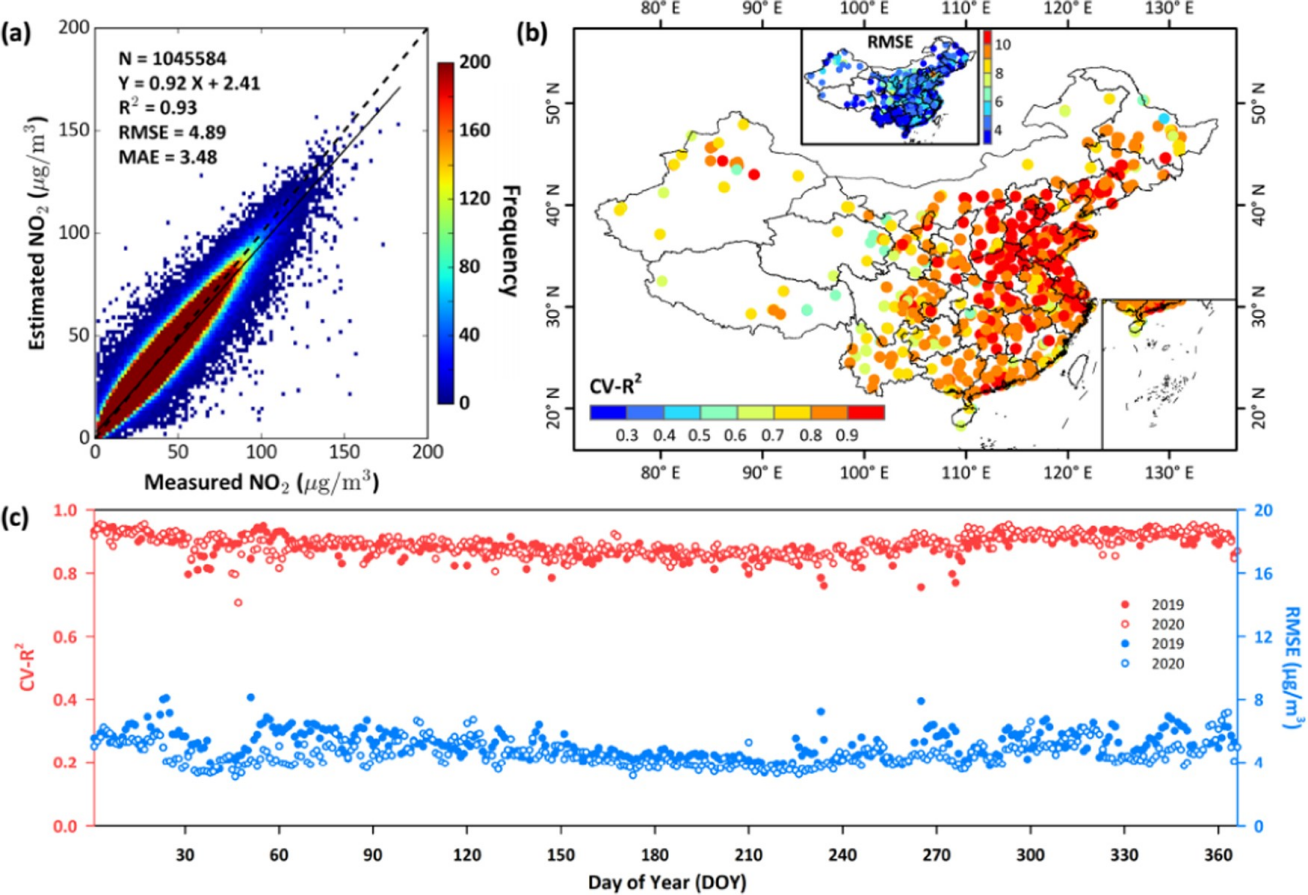
Out-of-sample cross-validation of daily ground-level NO_2_ estimates (μg/m^3^) (a) over the whole of China, (b) at each monitoring station, and (c) for each day of 2019 (filled dots) and 2020 (unfilled dots).

For ground-level NO_2_ data, we first evaluate the estimation accuracy of the developed SWDF model based on the out-of-sample CV approach at different spatiotemporal scales in China (Figure 2). Daily surface NO_2_ estimates (number of estimates, *N* = 1,045,584) are highly consistent with ground measurements (CV*-R*^2^ = 0.93), showing low uncertainties, with an average RMSE of 4.89 μg/m^3^ and mean absolute error (MAE) of 3.48 μg/m^3^ during 2019–2020 over the whole of China (Figure 2a). However, our surface NO_2_ estimates tend to be biased low but not much (slope from linear regression = 0.92). This is mainly because the smaller number of data samples in the case of heavy pollution can affect the model training. Also, satellite tropospheric NO_2_ columns under heavily polluted conditions are easily underestimated. Our model works well (e.g., CV-*R*^2^ = 0.91–0.94, RMSE = 5.2–5.5 μg/m^3^) in three typical urban agglomerations (Table S2). It also performs well at the individual-site scale, especially in East China (e.g., CV-*R*^2^ > 0.9, RMSE < 4 μg/m^3^). In general, approximately 83 and 85% of the monitoring stations across mainland China have high CV-*R*^2^ values greater than 0.8 and low RMSE values of less than 6 μg/m^3^, respectively (Figure 2b). The model is also relatively stable and less affected by time changes, and it can well estimate the time series of surface NO_2_ concentrations on most days (Figure 2c).

We next examine the predictive ability of our model according to the out-of-city CV approach at varying spatiotemporal scales in China (Figure S2). The model can well predict daily NO_2_ concentrations at locations in China where there are no ground-based measurements (i.e., CV-*R*^2^ = 0.71, RMSE = 9.95 μg/m^3^, and MAE = 7.4 μg/m^3^; Figure S2a). It also shows a strong predictive ability at regional scales (e.g., CV-*R*^2^ = 0.7–0.77, RMSE = 9.5–11.1 μg/m^3^; Table S2). Predictions are also reliable with small uncertainties at ∼84% of the stations (i.e., CV-*R*^2^ > 0.5, RMSE < 12 μg/m^3^), especially those located in eastern China where the observation network has a high station density (Figure S2b). The model can capture daily variations of surface NO_2_ in areas without available surface observations on most days in the years considered (Figure S2c). Compared to the estimation accuracy, the decline in predictive accuracy is mainly due to huge differences in the level of economic development among Chinese cities, especially in the eastern and western regions, which is closely related to NO*_x_* emissions.

### 3.2 Spatiotemporal Characteristics of Surface NO_2_

#### 3.2.1 ChinaHighNO_2_ Dataset

Using the SWMET model, we have generated a daily, full-coverage (100%), high-resolution (1 km), and high-quality ground-level NO_2_ dataset across mainland China (i.e., ChinaHighNO_2_) for the years 2019 and 2020, one of the series of ChinaHighAirPollutants (CHAP) dataset. Monthly and annual surface NO_2_ datasets are also synthesized by averaging daily data. These temporal surface NO_2_ composites (*N* = 35,496, and 2958, respectively) agree well with ground observations (*R*^2^ = 0.95 and 0.96, respectively), with low uncertainties (e.g., RMSE = 3.11 and 2.35 μg/m^3^, respectively) in China. These results further illustrate that our ChinaHighNO_2_ dataset can be used to study spatiotemporal variations in surface NO_2_ exposure across China.

**Figure 3:**
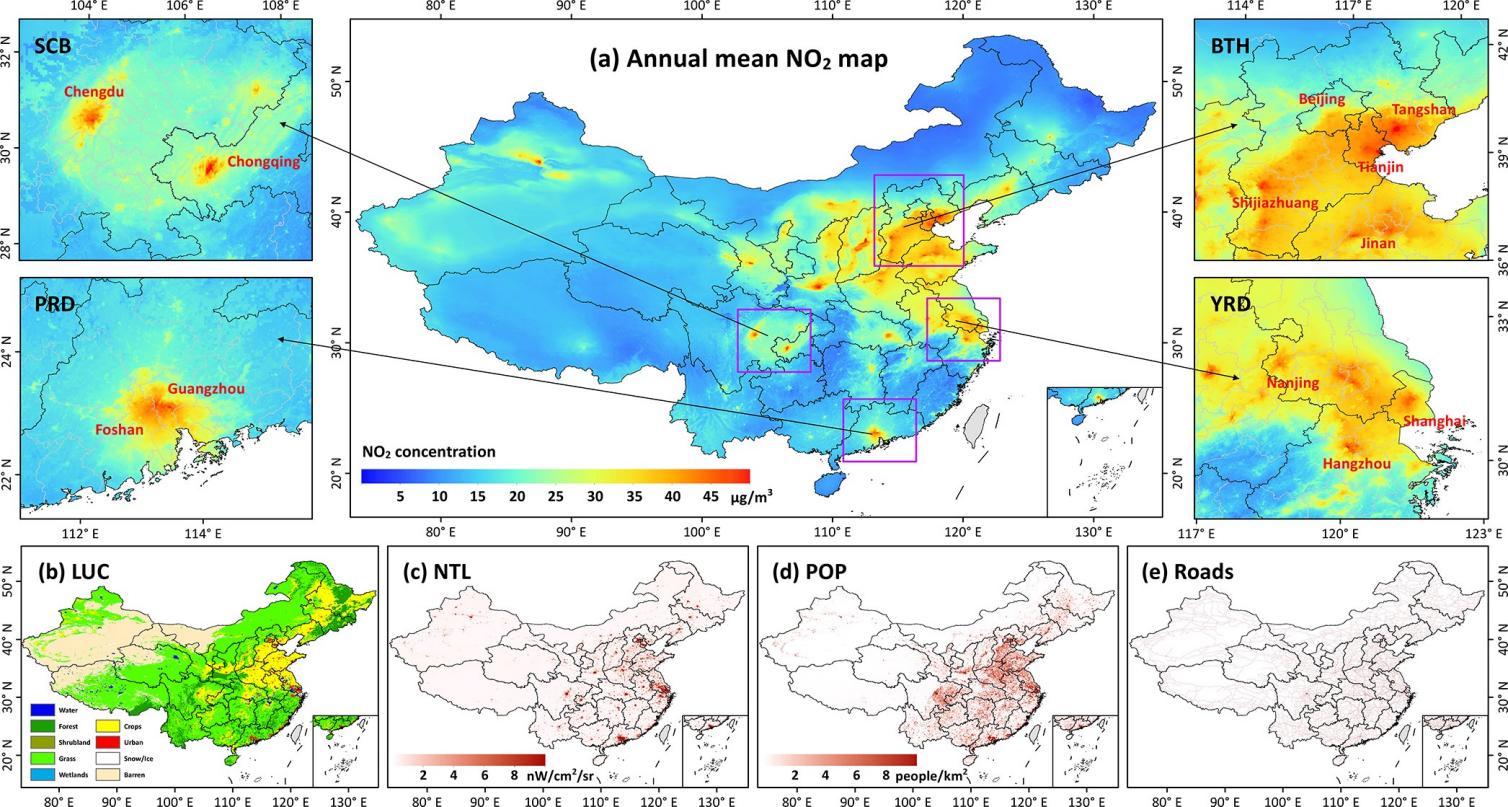
Spatial distributions of annual mean (a) national and regional (zoomed-in subplots) ground-level NO_2_ concentrations (μg/m^3^), (b) land-use cover (LUC), (c) nighttime lights (NTL), (d) population (POP), and (e) roads in China. Regions shown in panel (a) are the Sichuan Basin (SCB), Beijing–Tianjin–Hebei (BTH), the Pearl River Delta (PRD), and the Yangtze River Delta (YRD).

Figure 3 shows the spatial distributions of national and regional annual mean surface NO_2_ concentrations during 2019–2020, and their potential influential factors across China. The annual mean NO_2_ concentration was 17.5 ± 6.5 μg/m^3^ in China. In general, urban and cropland areas (red and yellow areas, respectively, in Figure 3b) corresponded to high surface NO_2_ concentrations, where high pollution levels (i.e., annual NO_2_ > 40 μg/m^3^) were mainly observed in central and eastern China regions with developed economies and concentrated populations (Figure 3c,d), e.g., the Beijing–Tianjin–Hebei (BTH) urban agglomeration and the Yangtze River Delta (YRD). In northwest China, the distribution of surface NO_2_ concentrations generally followed the distributions of populations and roads, suggesting that the sources of NO_2_ were mainly from anthropogenic and transportation emissions (Figure 3d,e). By contrast, southwest and northeast China had low NO_2_-pollution levels due to limited human activities in those regions. However, there are still positive biases in surface NO_2_ estimates in the vast cleaner areas (e.g., the Taklimakan Desert and Tibet), mainly because oxidized nitrogen species are present, leading to uncertainties in surface measurements.^43,44^ It is also worth noting that for any machine-learning (ML), especially deep-learning (DL), method, having an adequate number of ground-based stations to provide training data is essential. Less than a certain number, the ML would not have enough samples to “learn”, which is especially the case in western China where the stations are very few, and they do not represent the vast area of uninhabited land. Fortunately, this issue does not affect our conclusions that are chiefly concerned with air quality in more populated regions in eastern China.

The ChinaHighNO_2_ dataset can provide detailed surface NO_2_ information at finer city levels due to its high 1 km spatial resolution. Tianjin, Tangshan, and Langfang are the three cities with the most severe surface NO_2_ exposure. Most of the top 30 polluted cities are located in traditional heavy industrial areas in China, e.g., the BTH region and Shandong and Henan provinces. Large amounts of pollution gases are emitted from manufacture plants (Figure S3a).^45^ In general, surface NO_2_ levels are positively correlated with the level of urban economic development and population density, as proxied by the logarithm of NTL (*R* = 0.64, *p* < 0.001)^21^ and the population number (*R* = 0.41, *p* < 0.001), respectively (Figure S3b). We then calculated surface NO_2_ concentrations between urban and suburban areas, segmented from harmonized NTL using the stepwise-partitioning framework,^46^ at all cities in China. Large urban–rural differences are seen (average relative difference = 28%), especially for the provincial capitals, accounting for more than one-third of the top 30 prefecture-level cities (Figure S4). Such differences are closely associated with the size of the urban area compared with the size of the rural area and the distribution and density of populations. For example, Urumqi and Xi’an in western China have small core urban areas located with the highest population density (Figure 3d), leading to greater urban/rural NO_2_ differences.

Surface NO_2_ concentrations varied significantly on a seasonal basis (Figure S5), where the highest surface NO_2_ occurred in winter, with an average value of 20.9 ± 8.9 μg/m^3^, especially in the BTH region (∼39.2 ± 13.0 μg/m^3^). The next highest surface NO_2_ concentrations occurred in autumn (average = 19.3 ± 7.4 μg/m^3^) followed by spring (average = 16.2 ± 6.2 μg/m^3^). The peak in winter results from the combustion of coal and fossil fuels for heating in northern China, emitting a large amount of NO*_x_*. NO_2_ concentrations were lowest in summer (average = 13.8 ± 4.1 μg/m^3^), especially in southern China. This was mainly due to faster chemical loss via the OH + NO_2_ + M reaction. Summertime and low latitudes generally have higher levels of solar radiation and OH, shortening the NO_2_ lifetime.

#### 3.2.2 Holiday and Weekly Effects

The effects of holidays in China, i.e., the Spring Festival (5–11 February 2019) and National Day (1–7 October 2019), on surface NO_2_ concentrations are first investigated (Figures S6 and S7). In general, the spatial patterns over time of our model-derived surface NO_2_ concentrations agree well with ground observations. Before the Spring Festival, surface NO_2_ first remained high until February 1 due to normal conditions in most areas in China, especially in northern China, dominated by heavy industry. During the holiday, surface NO_2_ concentrations declined rapidly in eastern China because most factories gradually closed down over time, greatly reducing anthropogenic emissions. The lowest level of NO_2_ concentration in the country occurred around 7 February. Toward the end of the holiday, factories began to reopen, and surface NO_2_ levels gradually picked up. After the lantern festival (19 February), surface NO_2_ concentrations returned to their normal levels.

Surface NO_2_ concentrations during the National Day holiday had a similar temporal trend, i.e., high before the holiday, gradually decreasing over time, reaching a minimum value around 4 October during the holiday, then gradually increasing, returning to their normal levels on 9 October. These two general rounds of change and recovery of surface NO_2_ for the two festivals lasted for about 4 and 2 weeks, respectively, which was closely related to anthropogenic emissions. However, significant decreases in NO_2_ concentration before the festivals (e.g., 30 January to 1 February, 25–27 September) or the sudden fluctuations over a short period (e.g., 17–21 February, 9–13 October) may have likely been due to changes in meteorological conditions.

**Figure 4:**
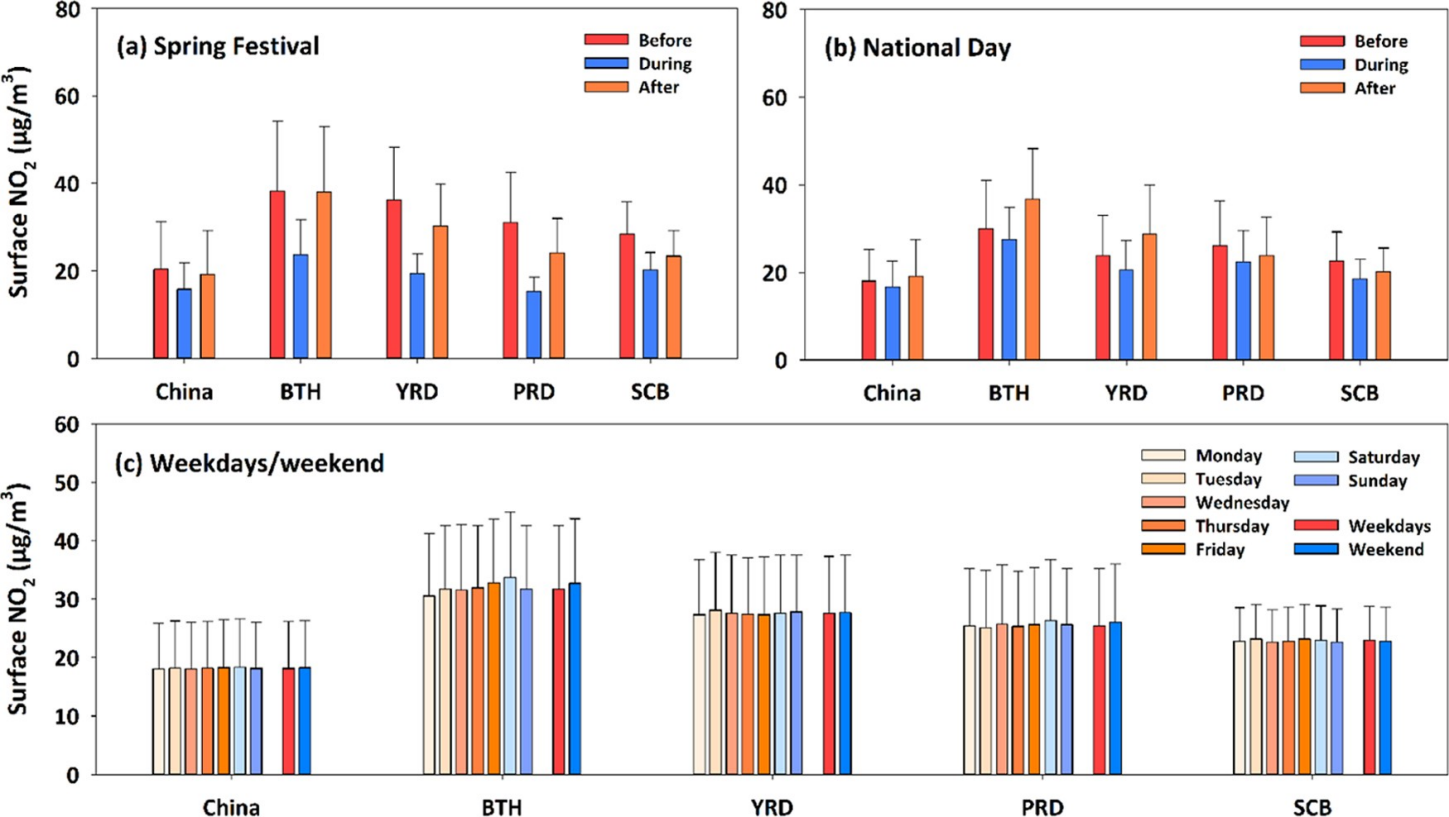
Comparison of average surface NO_2_ concentrations (μg/m^3^) before, during, and after (a) Spring Festival and (b) National Day holidays, and (c) during weekdays and the weekend in China and four typical regions. BTH, YRD, PRD, and SCB stand for Beijing–Tianjin–Hebei, Yangtze River Delta, Pearl River Delta, and Sichuan Basin, respectively.

On the national and regional levels, surface NO_2_ concentrations during the Spring Festival and National Day holidays were much lower by 22 and 14% than those before and after these holidays in China, respectively. In the BTH and YRD regions, in particular, maximum relative differences reached above 60 and 39%, respectively, illustrating strong holiday effects on surface NO_2_ concentrations (Figure 4a,b). Surface NO_2_ concentrations showed a slowly increasing trend on weekdays, decreasing by ∼6% on Sundays in BTH. In the other three regions and over the whole of China, surface NO_2_ concentrations stayed at a relatively stable level, with small, irregular changes (Figure 4c). Overall, differences in surface NO_2_ concentrations in China and in each region between weekdays and the weekend were small, within ±1 μg/m^3^. This differs from other parts of the world, such as Europe and the United States, where surface NO_2_ is much higher on weekdays than on weekends.^47−49^ This is mainly attributed to differences in economic production activities, e.g., factories in China operate on a continuous schedule, leading to continuous emissions.

Although similar results can be concluded from ground measurements on holidays (Figure S8a,b), larger amplitudes are observed in surface NO_2_ changes before and after the holidays (2.8–3.7 times larger, especially over the whole of China and SCB). The main reason is that stations are unevenly distributed and mainly located in cities, reflecting NO*_x_* emissions in urban areas. By contrast, satellite tropospheric NO_2_ data capture weaker or even opposite holiday effects (Figure S9a,b). Similar weak weekday/weekend differences are observed from all data sources. With respect to NO_2_ changes through the week, our results are more consistent with ground measurements, while tropospheric NO_2_ column changes tend to be irregular (Figures S8c and S9c). The full coverage of our dataset makes up for the spatial and data heterogeneities inherent to surface observations and more accurately describes changes in surface NO_2_ in the region.

#### 3.2.3 Surface NO_2_ Changes During the COVID-19 Pandemic

**Figure 5:**
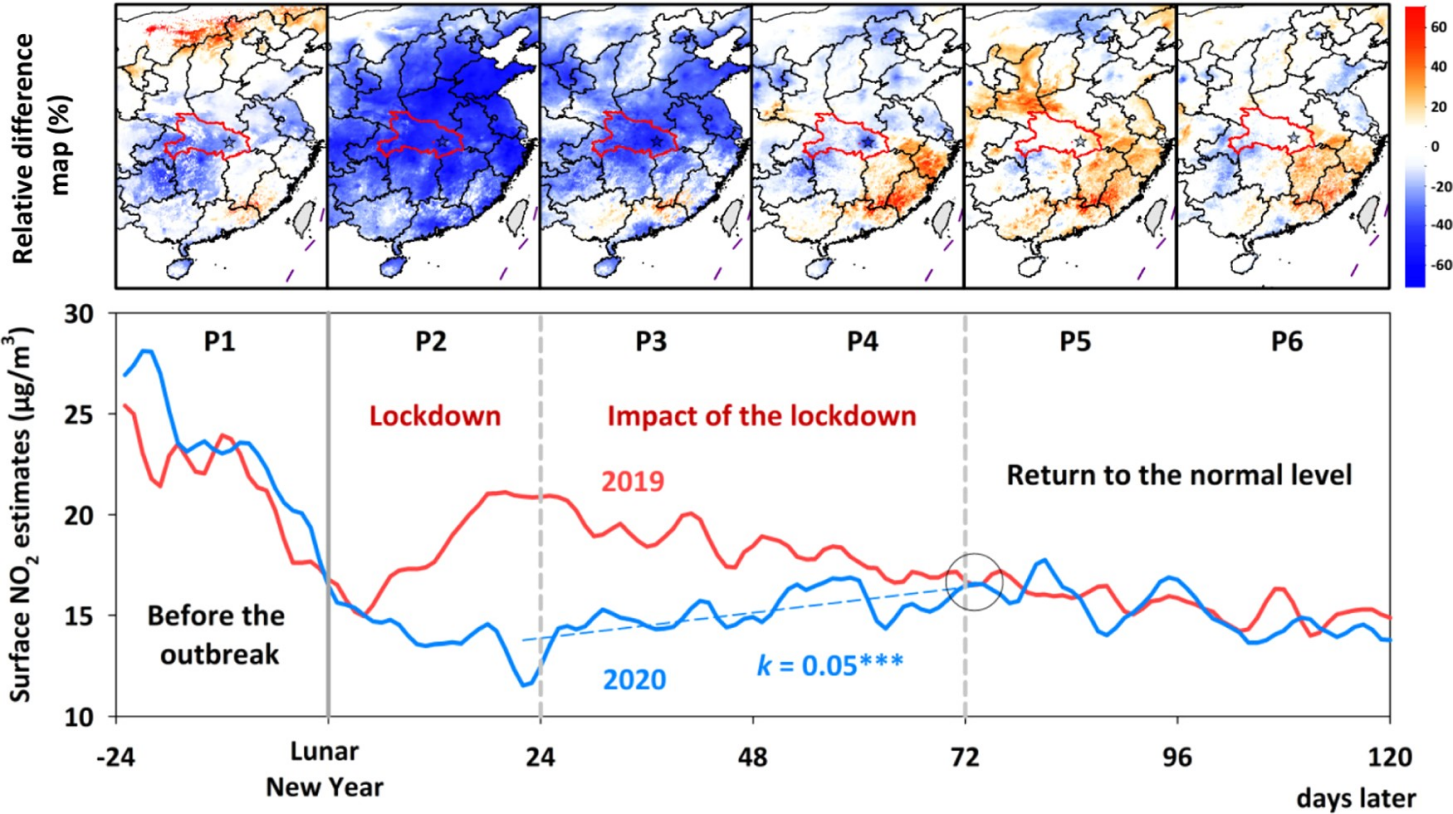
Time series of the 3-day moving average of daily surface NO_2_ concentrations (μg/m^3^) in China (bottom panel) and the relative difference (%) in surface NO_2_ concentrations (μg/m^3^) between 2020 and 2019 (top panel) during six periods (i.e., P1–P6) in eastern China before and after the Lunar New Year. The red border and star in the top panel indicate Hubei Province and Wuhan City, respectively. The gray circle in the bottom panel highlights when the surface-measured NO_2_ concentration from 2020 reached the 2019 historical level. The dashed blue line shows the linear trend for observations during the period experiencing the impact of the lockdown in 2020. The slope (*k*) is given, and the three asterisks indicate *p* < 0.001.

Surface NO_2_ variations related to the COVID-19 epidemic,^50−52^ that broke out in Wuhan, Hubei province, China, are investigated. Figure 5 shows the time series of daily ground-level NO_2_ concentrations and relative changes between 2020 and 2019 during six periods (denoted as P1–P6) before and after the Lunar New Year in eastern China. Before the outbreak (P1), surface NO_2_ concentrations changed little in most areas of eastern China but began to decrease in Hubei province and surrounding areas. During the lockdown (P2), surface NO_2_ concentrations dropped sharply, with relative changes greater than 60% over nearly all of eastern China. This was mainly due to the cessation of industrial production and human activities, significantly reducing NO*_x_* emissions.^53,54^ Previous studies based on ground-based measurements and satellite estimates have also reported this phenomenon.^51,55^ The lockdown had a strong and sustained impact on surface NO_2_ concentrations (relative changes > 30%) until the 48th day after the Lunar New Year (P3). After P3, the impact gradually decreased in most areas (P4) due to increasing anthropogenic emissions as the epidemic was gradually controlled and cities lifted bans.^53^ During P5 and P6, surface NO_2_ concentrations gradually recovered to their historical levels, with relative changes within ±20% in most areas of East China, including severe epidemic areas (e.g., Hubei province) and even slightly higher in some low-risk areas, e.g., southeast China, indicating that human life had returned to normal. Note that there were also some significant hotspots, e.g., in Henan and Fujian provinces, in different periods. These areas are mainly forested and densely vegetated areas with little or no population (Figure 3b,d) and very low surface NO_2_ levels, where small changes will lead to large relative differences.

Figure S10 compares variations in tropospheric NO_2_ columns and ground NO_2_ measurements during the COVID-19 pandemic. A similar temporal trend was found, namely, that NO_2_ significantly decreased during the lockdown,^56^ reaching a minimum value around the 22nd day after the Lunar New Year and rising steadily over time after the lockdown ended. However, noticeable differences were seen in the timing of the NO_2_ recovery. Tropospheric NO_2_ in 2020 first intersected with the historical level of 2019 (highlighted by the black circle in Figure S10b) on the 52nd day after the Lunar New Year (16 March 2020), with both time series generally following the same trend during P5 and P6. For surface-measured NO_2_ concentrations, this intersection with the historical level of 2019 (highlighted by the black circle in Figure S10a) occurred around the 72nd day after the Lunar New Year (5 April 2020), also seen in our modeled results (Figure 5). The duration of the impact of the lockdown on the tropospheric NO_2_ column was about 1.7 times shorter than that on surface NO_2_. Large differences were also found in the speed of NO_2_ recovery after lockdown. The slope of the trend for observations (Figure S10a) was the steepest (∼0.2 μg/m^3^/day, *p* < 0.001), four times that of ours (Figure 5), while that for the tropospheric NO_2_ column (Figure S10b) was the least steep (∼0.02 μg/m^3^/day, *p* < 0.001). This is mainly due to the difference in spatial representation, i.e., observations represent the change in NO_2_ in urban areas, while the tropospheric NO_2_ column represents the whole troposphere. Such a difference suggests that surface NO_2_ can better represent changes in NO*_x_* emissions at the local scale compared to the tropospheric NO_2_ column. The potential reasons may be that NO_2_ is directly related to the emission strength as a result of the relatively short lifetime (hours near the surface). Therefore, surface NO_2_ is more sensitive to anthropogenic emissions, e.g., fuel combustion, urban automobile exhaust, and industrial production, often occurring at the ground level. By contrast, tropospheric NO_2_ contains more information coming from complex sources that are smeared over large areas and can be further affected by convection or diffusion like transport, chemistry, and atmospheric lightning.

#### 3.2.4 Surface NO_2_-Pollution Exposure Risk

The unique advantage of the full coverage of our ChinaHighNO_2_ dataset is the ability to assess the daily surface NO_2_ exposure risk, i.e., exceedance of the national air quality standard (i.e., daily NO_2_ = 80 μg/m^3^), at every location across China in 2019 and 2020, separately (Figure S11). In 2019, most areas of China met the acceptable ambient NO_2_ standard year-round, except for key urban agglomerations, i.e., BTH, YRD, and the Pearl River Delta. In particular, core urban areas of the capitals and megacities in the main provinces (e.g., Tianjin, Shijiazhuang, Jinan, Taiyuan, Xi’an, Wuhan, Shanghai, and Foshan) had a high exposure risk, i.e., the percentage of days in 2019 not meeting the acceptable ambient NO_2_ standard exceeded 10%. A similar spatial pattern was observed in 2020, the area with an ambient NO_2_ exposure risk expanding into the YRD. Overall, in 2020, the probability of NO_2_-pollution occurrence declined across mainland China, especially in three typical urban agglomerations, mainly due to the impact of the epidemic.

### 3.3 Discussion

#### 3.3.1 Advantage of the New Model

Unlike traditional “black box” deep-learning models, the tree-based DF can be more physically interpretable, allowing the assessment of the importance of each input variable to model construction (Figure S12). Tropospheric NO_2_ is dominant, with the highest importance score (>31%), followed by the modeled surface NO_2_ (importance score = 15%). Spatial and temporal terms account for 14 and 10%, respectively, highlighting the importance of spatiotemporal information to air pollution modeling. Meteorological conditions also play key roles, especially RH and TEM, with a cumulative importance score near 20%. Variables related to the surface and population also have important impacts, contributing ∼5% each.

#### 3.3.2 Comparison of Different NO_2_ Products

Only a few model studies developed focused on near-surface NO_2_ in China have performed gap filling. Our model performs better than others,^24,25^ or is compatible,^20^ in terms of overall accuracy (Table S3). Our results should be superior to traditional interpolation methods (e.g., inverse distance weighting and kriging), especially in areas with a complex terrain with rapidly varying land cover and topography.^52^ Our gap filling is smoother with less noise compared to FSDN-filling results (e.g., Figure 8 in Li and Wu, 2021)^20^ that neglect tropospheric model simulations, especially spatiotemporal autocorrelations of air pollution.

OMI tropospheric NO_2_ products were first used to derive ground-level NO_2_ concentrations at coarse spatial resolutions from the original ∼0.25°^15,17,18,57^ to direct resampling of ∼0.125^16,19^ or ∼0.1°^14^ (Table S4). Later, the resolutions of surface NO_2_ estimates improved to ∼0.05^24,58^ and ∼0.025°^25^ using the newly launched TROPOMI satellite. Our study further improved the spatial resolution to 1 km using TROPOMI NO_2_ retrievals via area-weighted downscaling, about 2.5–25 times higher than previous studies. Note that another study also generated 1 km surface NO_2_ data but relied on the much coarser OMI NO_2_ information as the main input.^20^ In terms of overall accuracy, our SWDF model is superior to traditional statistical regression models (e.g., GTWR,^14^ LUR,^16^ UK&SBM,^19^ and GTWR-SK^25^) and popular machine- and deep-learning models (e.g., RF-K, RF-SK,^18^ FSDN,^20^ LightGBM,^24^ BME,^57^ and XGBoost^58,59^), improving the CV-R^2^ by 9–55% and reducing the RMSE by 23–64%. The 1 km ground-level NO_2_ dataset represents a substantial improvement over existing surface NO_2_ data in China, potentially valuable for nitrogen-cycle and health-related studies, especially in urban areas.

## Supporting Information Available

Additional quality control (Test S1); tropospheric NO_2_ gap filling (Test S2); ground-level NO_2_ estimation (Test S3); spatial coverage of available daily (a) OMI and (b) TROPOMI tropospheric NO_2_ retrievals across China (Figure S1); and summary of the data sources used in this study (Table S1) (PDF)

## Notes

The ChinaHighNO_2_ dataset is available at https://doi.org/10.5281/zenodo.4571660, and the ChinHighAirPollutants (CHAP) dataset is available at https://weijing-rs.github.io/product.html.
