# Supplementary material for: Ground-Level NO2 Surveillance from Space Across China for High Resolution Using Interpretable Spatiotemporally Weighted Artificial Intelligence
Source: Environ Sci Technol. 2022 Jun 29;56(14):9988–98. doi: 10.1021/acs.est.2c03834 (PMC9301922; doi:10.1021/acs.est.2c03834)
Supplement: Supplementary file 1 — es2c03834_si_001.pdf [file es2c03834_si_001.pdf]

Supporting Information for

Ground-level NO_2 surveillance from space across China for high resolution using interpretable spatiotemporally weighted artificial intelligence

Jing Wei[1,2*#], Song Liu[3#], Zhanqing Li[2*], Cheng Liu[4], Kai Qin[5], Xiong Liu[6], Rachel T. Pinker[2], Russell R. Dickerson[2], Jintai Lin[7], K. F. Boersma[8,9], Lin Sun[10], Runze Li[11], Wenhao Xue[12], Yuanzheng Cui[13], Chengxin Zhang[4], Jun Wang[1*]

1. Department of Chemical and Biochemical Engineering, Iowa Technology Institute, Center for Global and Regional Environmental Research, University of Iowa, Iowa City, IA 52242, USA
2. Department of Atmospheric and Oceanic Science, Earth System Science Interdisciplinary Center, University of Maryland, College Park, MD 20742, USA
3. School of Environmental Science and Engineering, Southern University of Science and Technology, Shenzhen 518055, China
4. Department of Precision Machinery and Precision Instrumentation, University of Science and Technology of China, Hefei 230026, China
5. School of Environment and Geoinformatics, China University of Mining and Technology, Xuzhou 221116, China
6. Atomic and Molecular Physics Division, Center for Astrophysics | Harvard & Smithsonian, Cambridge, MA 02138, USA
7. Laboratory for Climate and Ocean-Atmosphere Studies, Department of Atmospheric and Oceanic Sciences, School of Physics, Peking University, Beijing 100871, China
8. Satellite Observations Department, Royal Netherlands Meteorological Institute, De Bilt 3731GA, the Netherlands
9. Meteorology and Air Quality Group, Wageningen University, Wageningen 6708PB, the Netherlands
10. College of Geodesy and Geomatics, Shandong University of Science and Technology, Qingdao 266590, China
11. Department of Civil and Environmental Engineering, University of California, Irvine, CA 92697, USA
12. School of Economics, Qingdao University, Qingdao 266071, China
13. College of Hydrology and Water Resources, Hohai University, Nanjing 210098, China

[#] Co-first authors who contributed equally to this study.

* Corresponding authors: weijing_rs@163.com; zli@atmos.umd.edu; jun-wang-1@uiowa.edu

Contents of this file

Texts S1-S3

Figures S1-S12

Tables S1-S4

Text S1: Additional quality control

For the vast barren and uninhabited land areas in Western China, e.g., southern Xinjiang and western Tibet, surface NO_2 concentrations are usually relatively low, especially at night and early morning. Considering that there are too few samples needed for the Deep Learning in western China, and a small number of potentially problematic samples could have a large impact on the model training, we defined a more objective approach to filter them via checking the diurnal variations in ground measurements: 1) First, we define these suburb clean sites in western China with little human activities using land use classification and population data; 2) for each day and each station, we count the percentage of hourly observations exceeding the daily (24-h average) NO_2 concentration limit (i.e., 40 μg/m^3); if the percentage is larger than 50%, the data from that site and that whole individual day is filtered out as outliers because such case is likely affected by instrument malfunction due to harsh natural conditions in western China. As the surface NO_2 concentrations are commonly lower than 40 μg/m^3 and the duration of diurnal NO_2 peak is typically shorter than 4 hours a day (when the anthropogenic activity is high and the planetary boundary layer is low),[1-3] our approach can effectively remove such potential outliers (e.g., for 28 Jan 2019).

Text S2: Tropospheric NO_2 gap filling

There are two iterations for tropospheric NO_2 gap filling using the SWMET model:

1) For the 1st iteration, available daily OMI tropospheric NO_2 retrievals (OMI_{TNO_2}) are regarded as the observations, and the missing values are predicted by regressing the SWMET model with spatially continuous auxiliary variables, including modeled tropospheric NO_2 ($Model_{TNO_2}$), six meteorological variables (including boundary layer height (BLH), relative humidity (RH), surface pressure (SP), temperature (TEM), 10-m u-component (WU) and v-component of winds (WV)), surface-related (i.e., Normalized Difference Vegetation Index (NDVI), and digital elevation model (DEM)) variables, and spatiotemporal terms (Ps and Pt):

$$OMI_{TNO_2} \sim f_{SWMET}(Model_{TNO_2}, BLH, RH, SP, TEM, WU, WV, DEM, NDVI, P_s, P_t) \quad (1)$$

2) For 2nd iteration, available daily TROPOMI tropospheric NO_2 retrievals (TRO_{TNO_2}) as the observations, along with the OMI tropospheric NO_2 predicted in the 1st iteration, modeled

tropospheric NO_2 ($Model_{TNO_2}$), and the same meteorological (i.e., BLH, RH, SP, TEM, WU, and WV), and spatiotemporal terms (Ps and Pt), are used to construct the second gap-filling model:

$$TRO_{TNO_2} \sim f_{SWMET}(OMI_{TNO_2}, Model_{TNO_2}, BLH, RH, SP, TEM, WU, WV, DEM, NDVI, P_s, P_t) \quad (2)$$

Text S3: Ground-level NO_2 estimation

There are a total of twenty-one features inputs to the spatiotemporally weighted deep forest (SWDF) model including the ground-based NO_2 measurements (Sur_{NO_2}), full-coverage TROPOMI ($FTRO_{TNO_2}$) and OMI ($FOMI_{TNO_2}$) tropospheric NO_2 data predicted in Test S1, modeled tropospheric ($Model_{TNO_2}$) and surface ($Model_{SNO_2}$) NO_2 data, NO_x emission, all eight meteorological ($Meteorology$) fields (i.e., BLH, evaporation (ET), precipitation (PRE), RH, SP, TEM, WU and WV), DEM, Land Use Type (LUC), NDVI, nighttime lights (NTL), and population distribution (POD), and spatiotemporal terms (Ps and Pt), can be expressed as:

$$Sur_{NO_2} \sim f_{SWDF}(FTRO_{TNO_2}, FOMI_{TNO_2}, Model_{TNO_2}, Model_{SNO_2}, NO_x, Meteorology, DEM, LUC, NDVI, NTL, POD, P_s, P_t), \quad (3)$$

There are three main steps during the model building: 1) first uses multi-Grained Scanning to extract features of different granularity of data; 2) then they are used as inputs to the Cascade Forest, in which each layer contains multiple forests constructed by random forest (RF) and completely-random trees (CRT); 3) last, the final output is combined from all layers' results using the Light Gradient Boosting Machine (LightGBM) model.

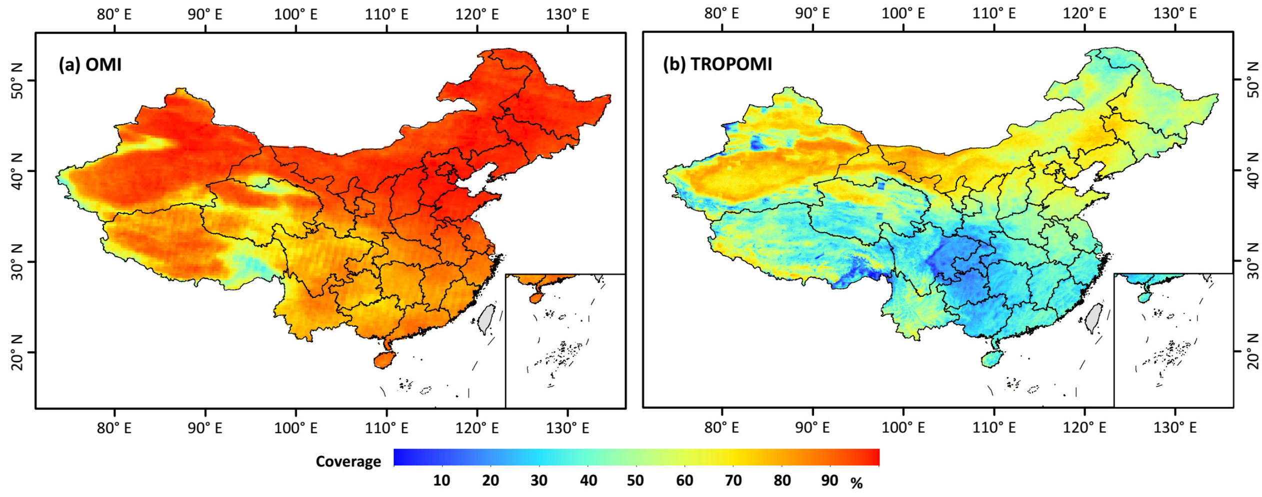

Figure S1. Spatial coverage of available daily (a) OMI and (b) TROPOMI tropospheric NO_2 retrievals across China.

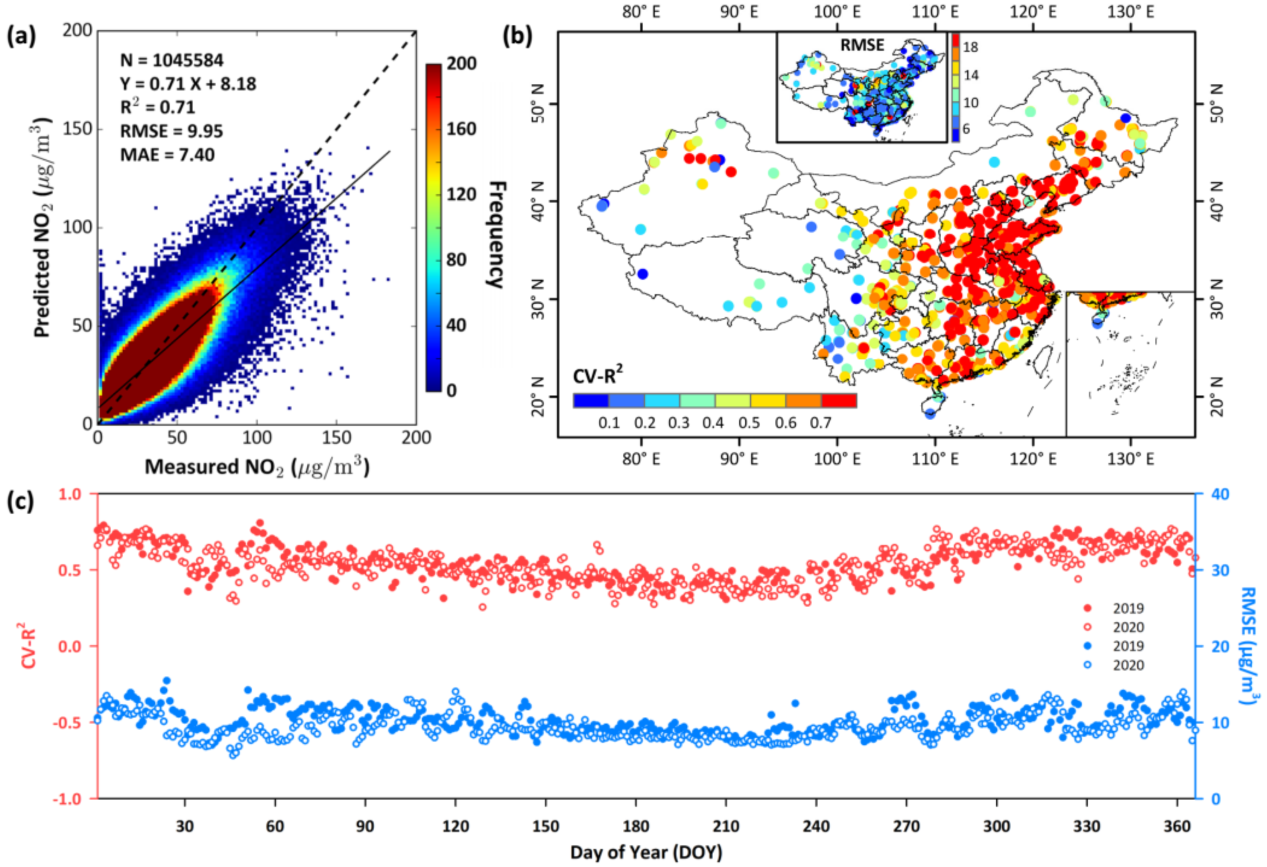

Figure S2. Same with Figure 2 but with the out-of-city cross-validation approach.

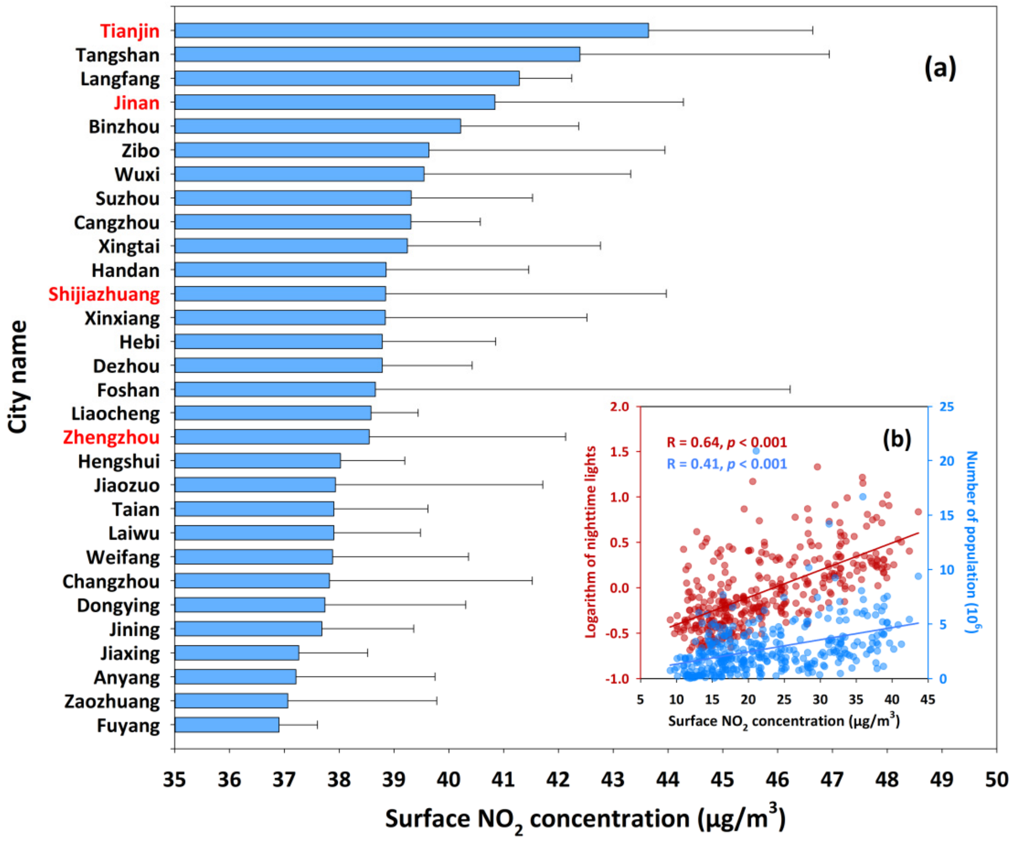

Figure S3. Sorted annual mean surface NO_2 concentrations ($\mu g/m^3$) at (a) top 30 cities (the red font indicates the provincial capital city of China), and their relationships with (b) the logarithm of nighttime lights (red) and number of population (blue) at all cities in mainland China.

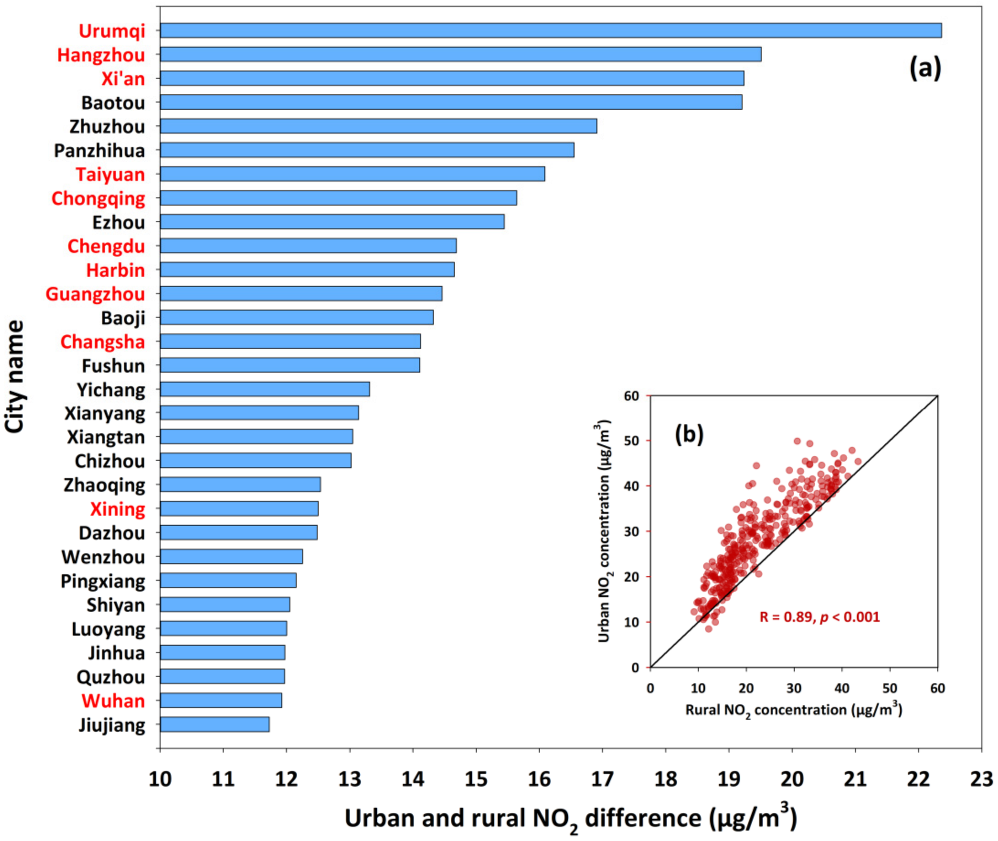

Figure S4. Urban-rural differences in annual mean surface NO_2 concentrations (μg/m^3) at (a) top 30 and (b) all cities in mainland China, where the red font indicates the provincial capital city of China.

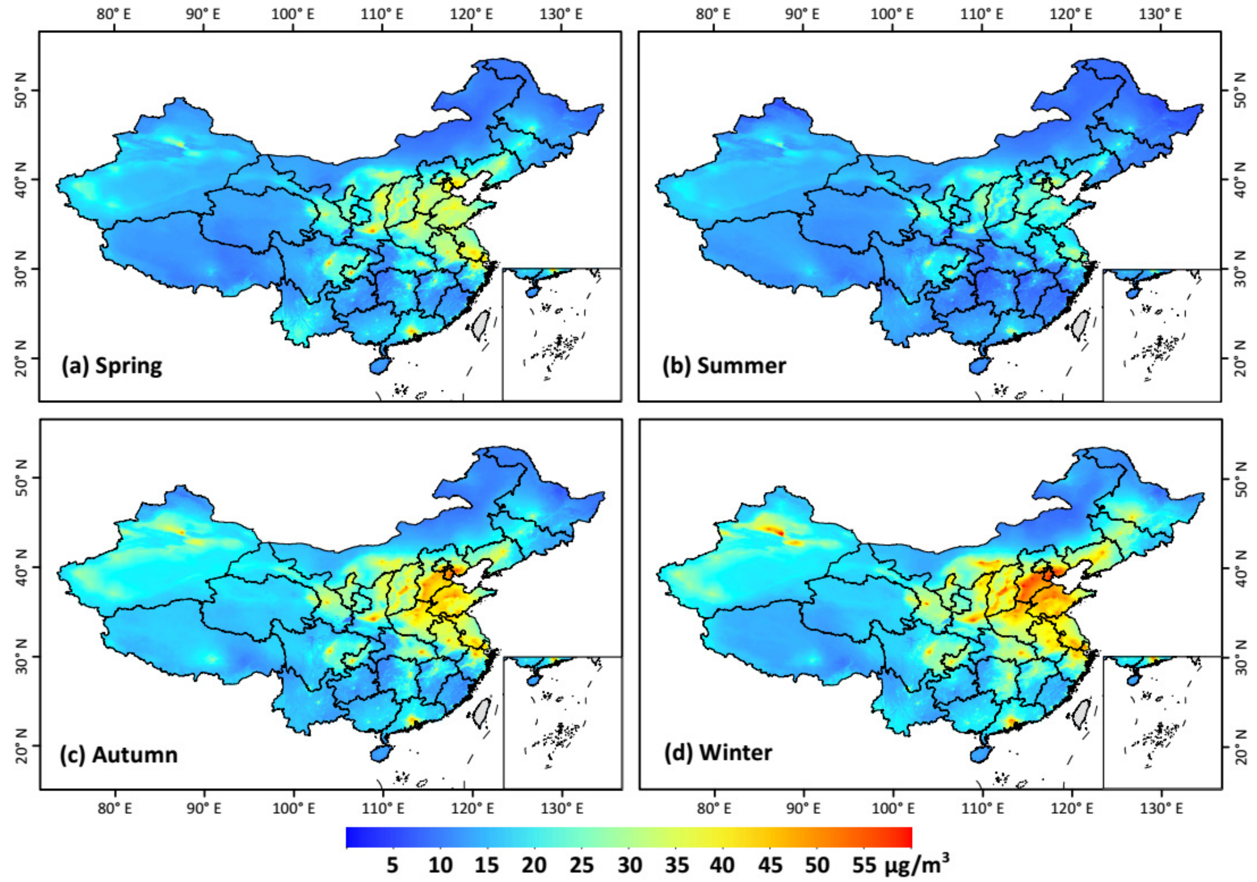

Figure S5. Seasonal mean ground-level NO_2 concentrations ($\mu g/m^3$) from 2019 to 2020 across China: (a) Spring, (b) Summer, (c) Autumn, and (d) Winter.

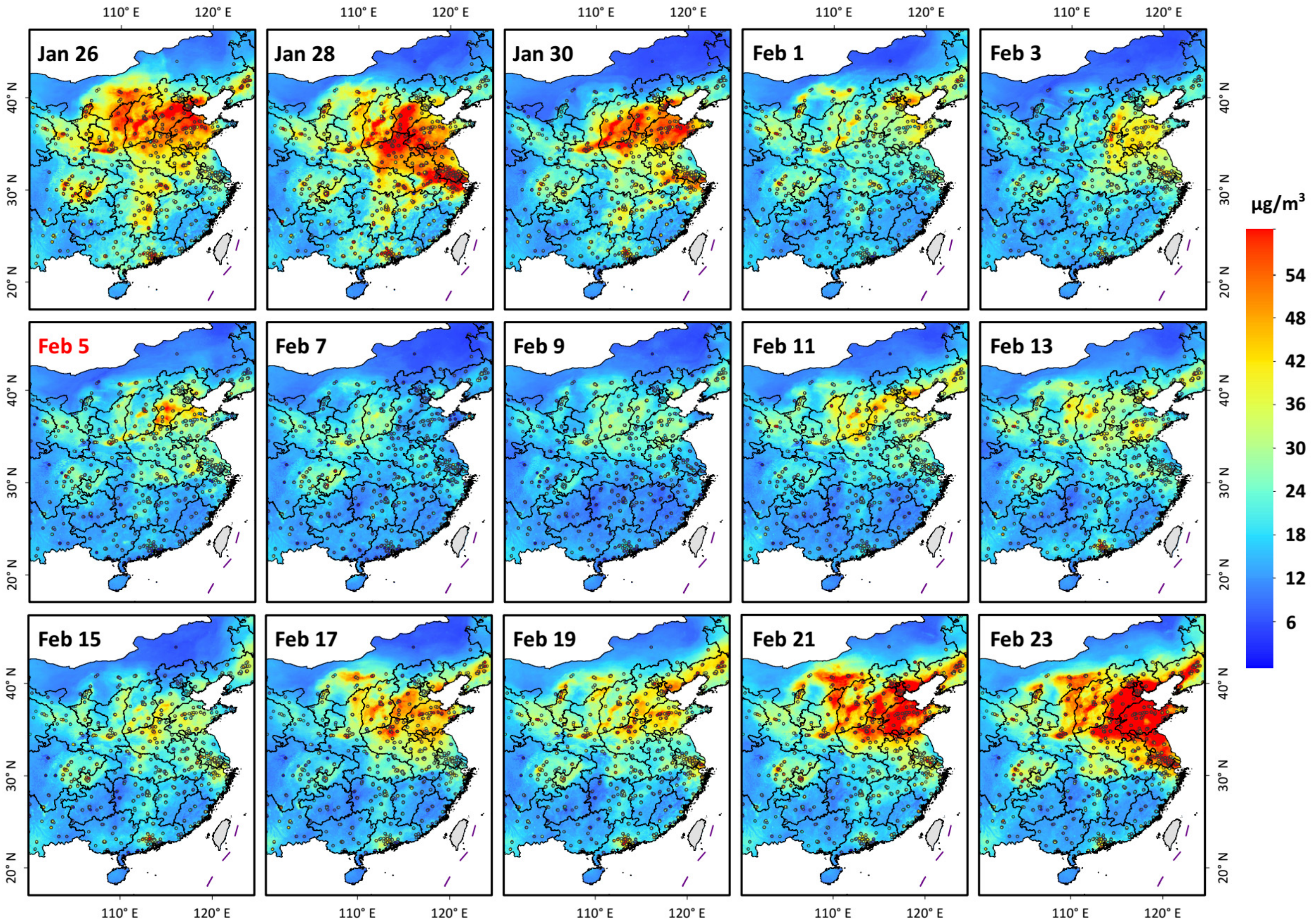

Figure S6. Temporal variations of our model-derived (background shading) and ground-measured (colored dots) daily ground-level NO_2 concentrations (μg/m^3) covering the Spring Festival (i.e., February 5–11) from January 26 to February 23 in 2019 across China, where the day of the Chinese Lunar New Year (i.e., February 5, 2019) is marked in red font.

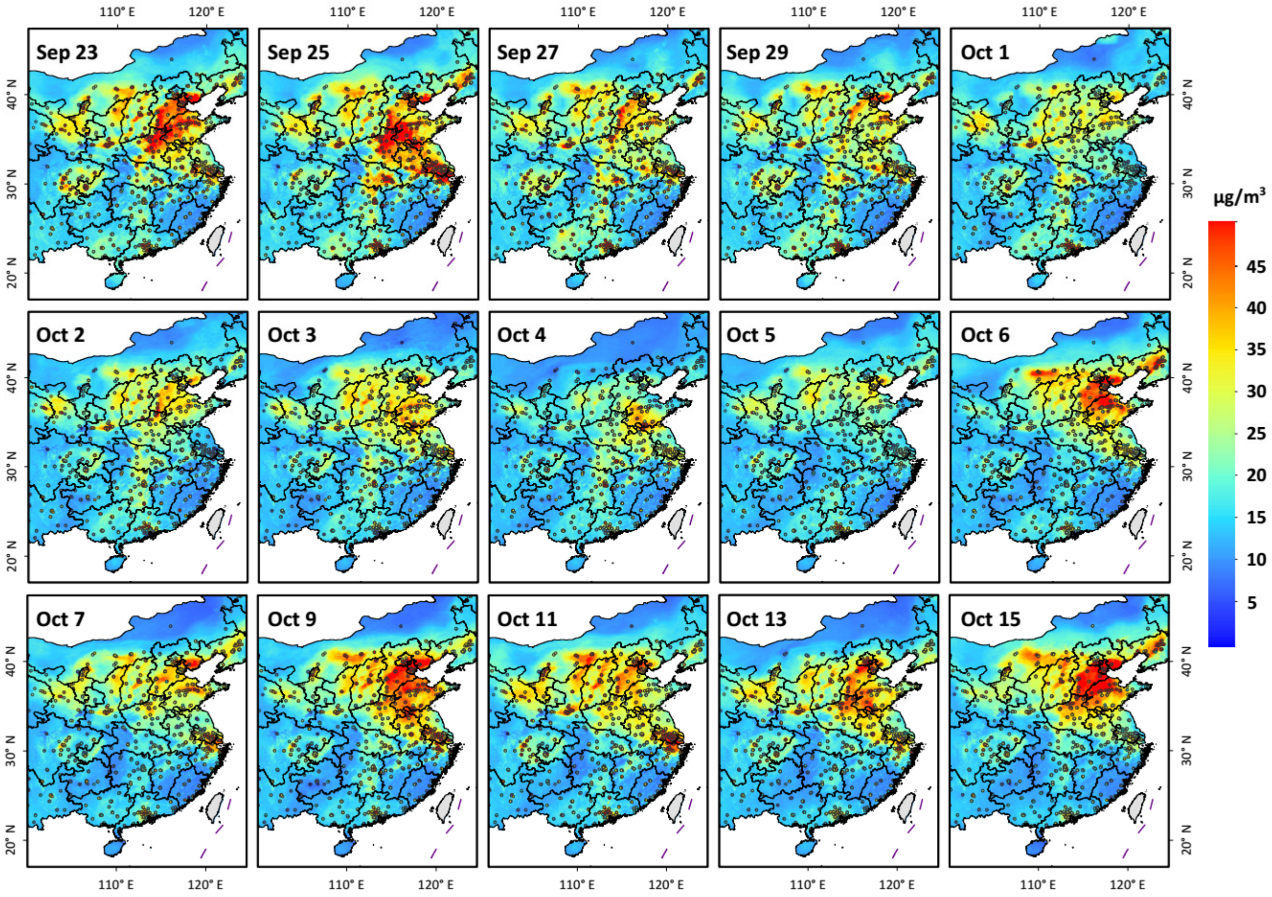

Figure S7. Temporal variations of our model-derived (background shading) and ground-measured (colored dots) daily ground-level NO_2 concentrations (μg/m^3) coving the National Day (i.e., October 1–7) from September 23 to October 15 in 2019 across China.

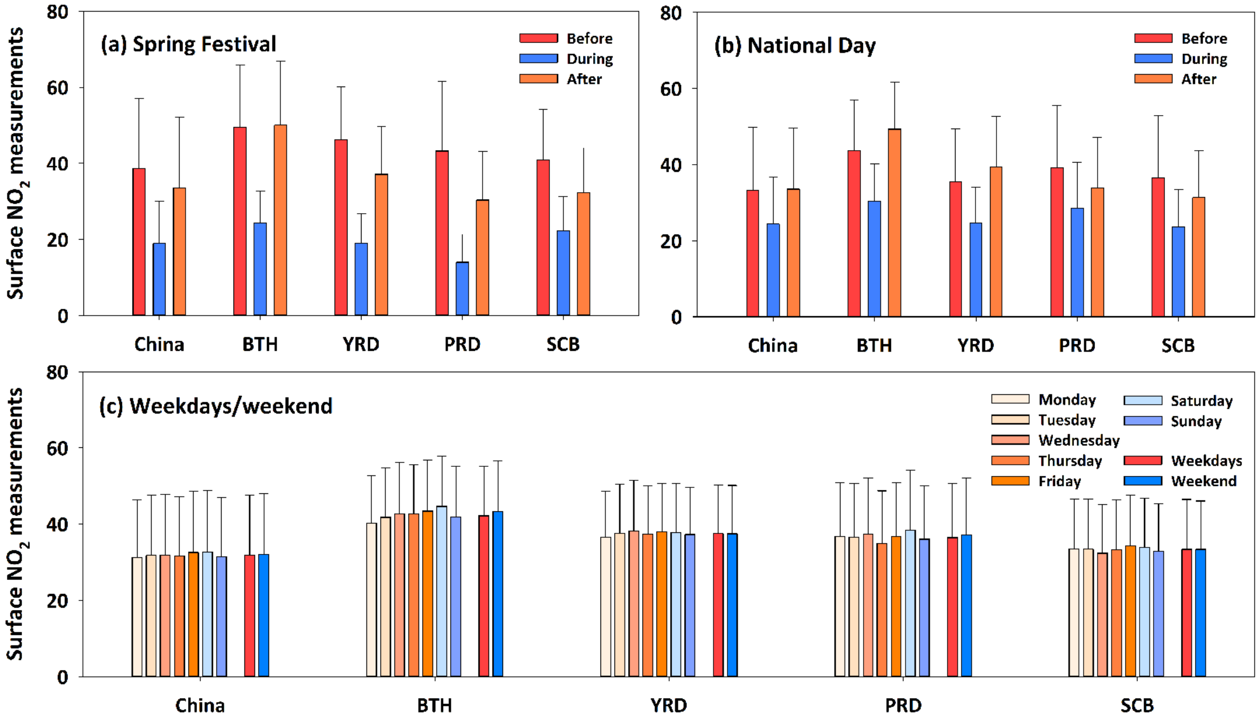

Figure S8. Comparison of average ground-based surface NO_2 measurements (μg/m^3) before, during, and after the (a) Spring Festival and (b) National Day holidays, and (c) during weekdays and weekends in China and four typical regions.

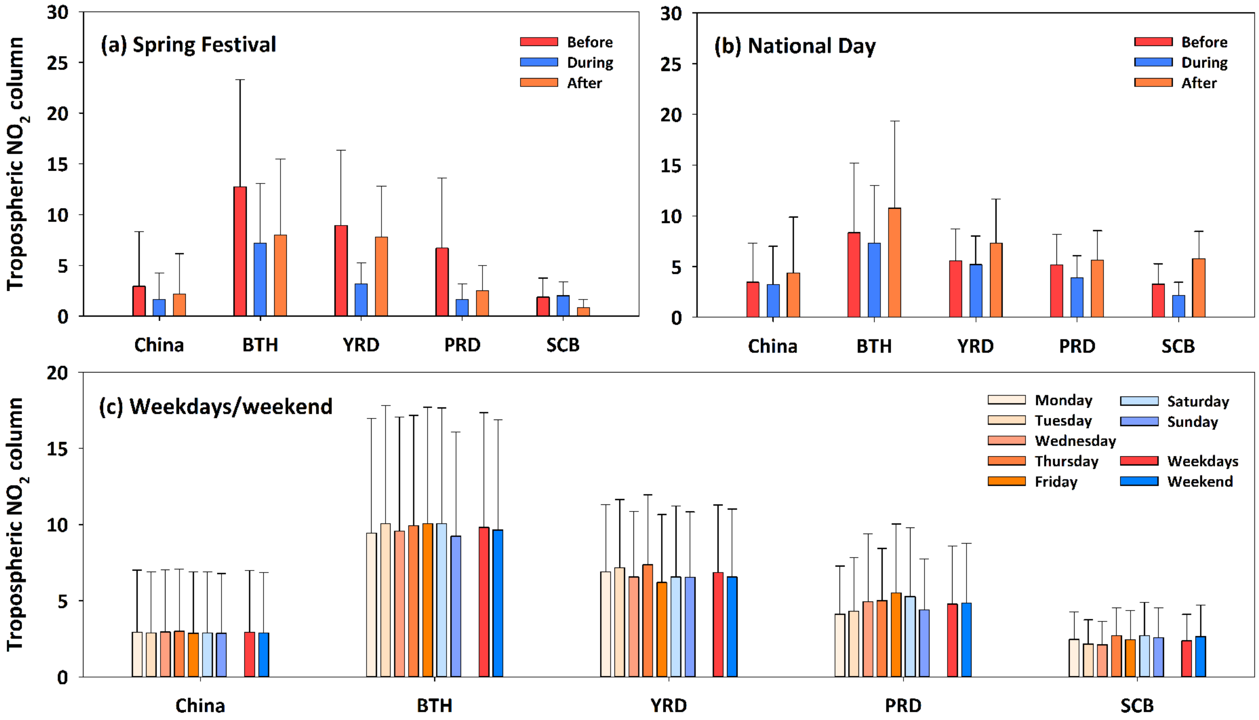

Figure S9. Comparison of average ground-based Tropospheric NO_2 column (10^{15} molec/cm^2) before, during, and after the (a) Spring Festival and (b) National Day holidays, and (c) during weekdays and weekends in China and four typical regions.

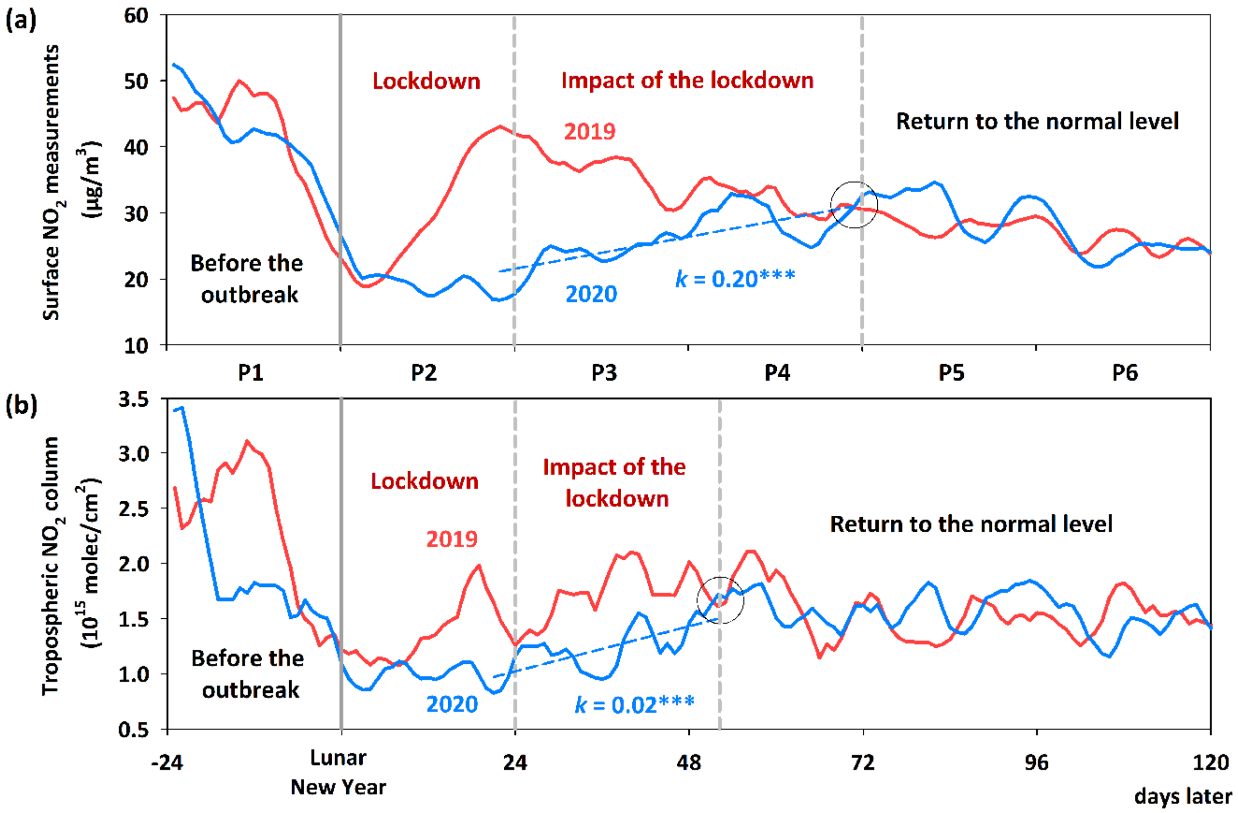

Figure S10. Comparison of time series of daily (a) surface NO_2 measurements ($\mu g/m^3$) and (b) TROPOMI tropospheric NO_2 columns (10^{15} molec/cm^2) in 2019 (red) and 2020 (blue) before and after the Lunar New Year in China. The grey circles highlight when surface-measured NO_2 concentrations and tropospheric NO_2 columns from 2020 reached 2019 historical levels. Dashed blue lines show the linear trends during the period experiencing the impact of the lockdown in 2020. The slope (k) is given, and the three asterisks indicate $p < 0.001$.

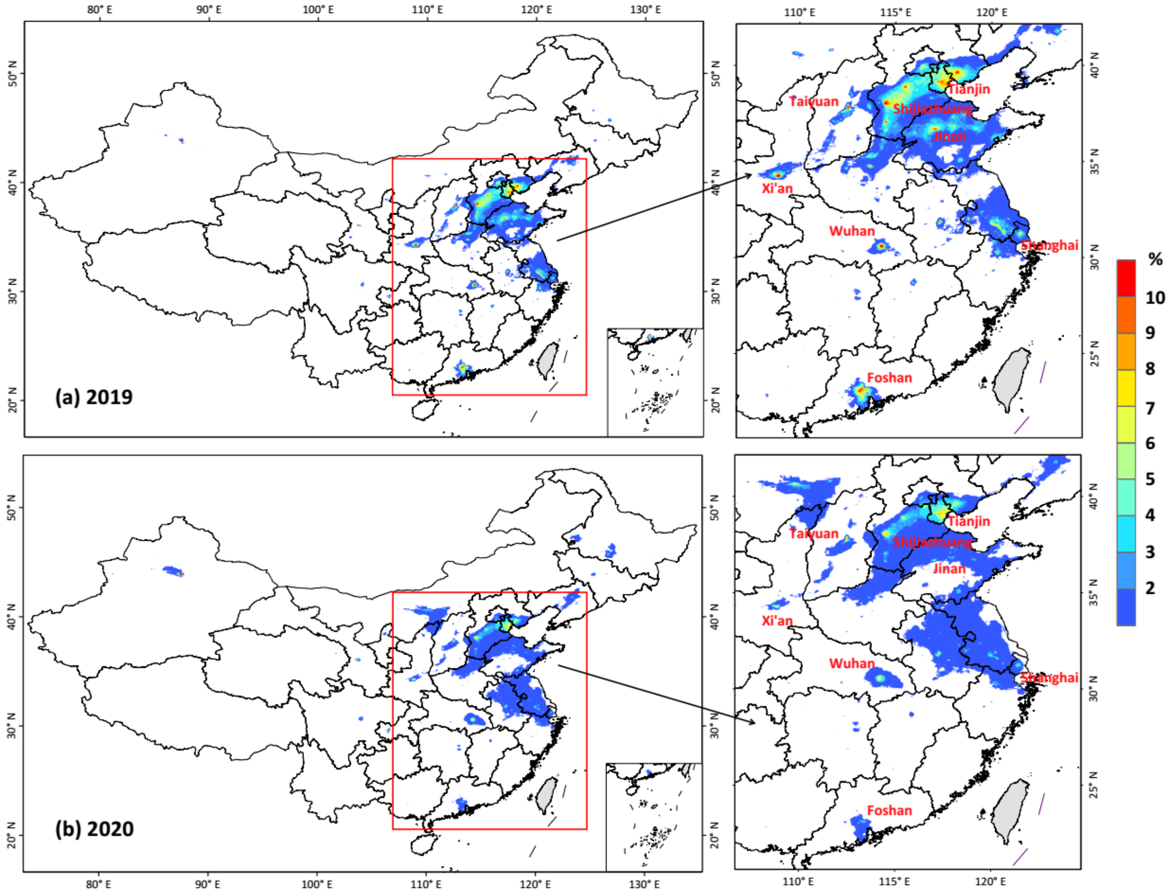

Figure S11. Spatial distributions of the percentage (%) of days exceeding the ambient NO_2 standard (i.e., daily NO_2 concentration = 80 μg/m^3) in 2019 and 2020 in China.

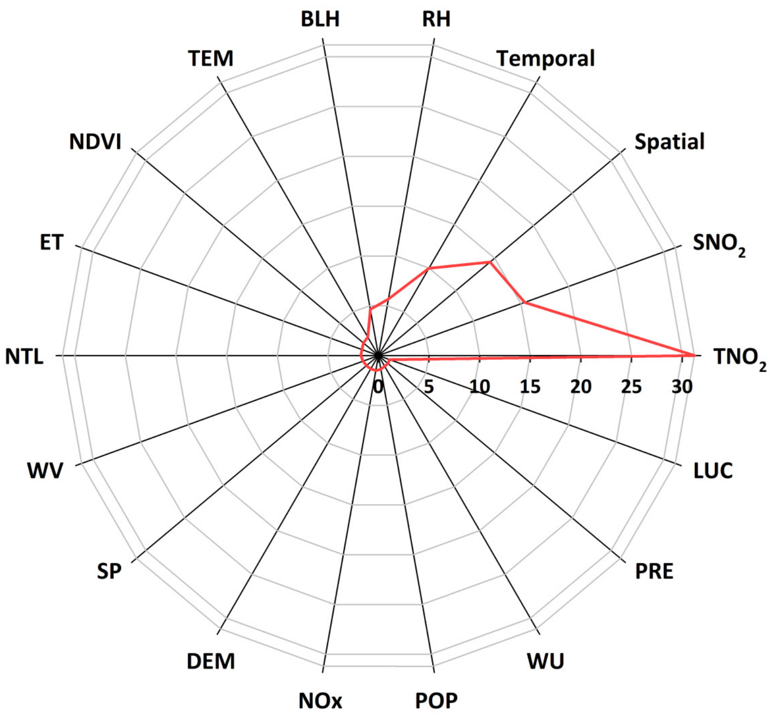

Figure S12. Radar plot of feature importance for ground-level NO_2 modeling.

Table S1. Summary of the data sources used in this study.

Variable	Description	Unit	Spatial Resolution	Temporal Resolution	Data Source
NO_2	Surface NO_2	μg/m^3	Point	Hourly	MEE
NO_2	tropospheric NO_2	molec/cm^2	1 km	Daily	USTC TROPOMI
	tropospheric NO_2	molec/cm^2	0.25°×0.25°	Daily	OMI
	tropospheric NO_2<br>Surface NO_2	molec/cm^2<br>μg/m^3	0.75°×0.75°	Daily	CAMS
NO_x	Nitrogen oxides	Mg/grid	0.1°×0.1°	Monthly	CAMS
LUC	Land cover type			Annual	MCD12
NDVI	Normalized difference vegetation index	-	0.05°×0.05°	Monthly	MOD13
DEM	Surface elevation	m	90 m	-	SRTM
NTL	Nighttime lights	nW/cm^2/sr	500 m	Monthly	VIIRS
POP	Population density	-	1 km	Annual	LandScan™
ET	evaporation	mm	0.1°×0.1°	Hourly	ERA5
PRE	Precipitation	mm			
SP	Surface pressure	hPa			
TEM	2-m air temperature	K			
WU	10-m u-component	m/s			
WV	10-m v-component	m/s			
BLH	Boundary layer height	m	0.25°×0.25°		
RH	Relative humidity	%			

MEE: Chinese Ministry of Environment and Ecology; USTC: University of Science and Technology of China.

Table S2. Out-of-sample (overall accuracy) and out-of-city (spatial prediction ability) cross-validation results of daily NO_2 estimates (μg/m^3) and predictions (μg/m^3) in the Beijing-Tianjin-Hebei (BTH), the Yangtze River Delta (YRD), and the Pearl River Delta (PRD) from 2019 to 2020 in China.

Region	Sample size	Overall accuracy			Spatial prediction ability		
	N	R^2	RMSE	MAE	R^2	RMSE	MAE
BTH	56,797	0.94	5.23	3.80	0.74	11.09	8.57
YRD	16,607	0.92	5.43	3.92	0.70	10.43	7.90
PRD	40,403	0.93	5.23	3.71	0.77	9.52	7.17

Table S3. Validation and comparison of tropospheric NO_2-gap filling methods in China

Gap-fill model	Relationship with			Literature
	Tropospheric NO_2		Ground NO_2	
	CV-R^2	CV-RMSE	R	
IDW & Time linear interpolation	–	–	0.59	Wu et al., 2021[4]
Exemplar-based algorithm	0.71–0.80	3.19–6.89	–	Wang et al., 2021[5]
Full residual deep networks	0.91–0.99	0.07–6.21	–	Li & Wu, 2021[6]
**SWMET**	**0.89–0.96**	**0.46–1.51**	**0.62**	**This study**

IDW: inverse distance weighting

Table S4. Comparison of model performances with previous NO_2 studies in China

Model	Spatial resolution	Cross validation		Main input predictor	Gap filling	Study region	Literature
		R^2	RMSE				
BME	0.25°	0.78	11.21	OMI NO_2	No	BTH	Jiang & Christakos, 2018[7]
RF-SK	0.25°	0.62	13.3	OMI NO_2	No	China	Zhan et al., 2018[8]
ERT	0.25°	0.72	9.20	POMINO NO_2	No	ECH	Qin et al., 2020[9]
	0.25°	0.70	9.42	OMI NO_2	No	ECH	
RF-K	0.25°	0.64	11.3	OMI NO_2	No	China	Dou et al., 2021[10]
XGBoost	0.125°	0.67	6.40	TROPOMI NO_2	No	China	Chi et al., 2022[11]
LUR	0.125°	0.78	-	OMI NO_2	No	China	Xu et al., 2019[12]
UK&SBM	0.125°	0.85	7.87	OMI NO_2	No	China	Chen et al., 2019[13]
GTWR	0.1°	0.60	-	OMI NO_2	No	ECH	Qin et al., 2017[14]
XGBoost	0.05°	0.83	7.58	TROPOMI NO_2	No	China	Liu, 2021[15]
LightGBM	0.05°	0.83	6.62	TROPOMI NO_2	Yes	China	Wang et al., 2021[5]
GTWR-SK	0.025°	0.84	6.70	TROPOMI NO_2	Yes	China	Wu et al., 2021[4]
FSDN	0.01°	0.82	8.80	OMI NO_2	Yes	China	Li & Wu, 2021[6]
**SWDF**	**0.01°**	**0.93**	**4.89**	**TROPOMI NO_2**	**Yes**	**China**	**This study***

BME: Bayesian maximum entropy; ERT: extremely randomized trees; FSDN: full residual deep networks; GTWR: geographically and temporally weighted regression; GTWR-SK: GTWR with spatiotemporal kriging; RF-K; LightGBM: Light Gradient Boosting Machine; LUR: land use regression; MEM: mixed effect model; RF-K: random forest integrated K-means; RF-SK: random forest integrated spatiotemporal kriging; SWDF: spatiotemporally weighted deep forest; UK&SBM: universal kriging & satellite-based model; XGBoost: extreme gradient boosting.

References

1. Yang, J.; Kang, S.; Ji, Z.; Yin, X.; Tripathee, L., Investigating air pollutant concentrations, impact factors, and emission control strategies in western China by using a regional climate-chemistry model. *Chemosphere* **2020,** *246*, 125767.
2. Zhao, Q.; He, Q.; Jin, L.; Wang, J., Potential Source Regions and Transportation Pathways of Reactive Gases at a Regional Background Site in Northwestern China. *Advances in Meteorology* **2021,** *2021*, 9933466.
3. Chen, P.; Yang, J.; Pu, T.; Li, C.; Guo, J.; Tripathee, L.; Kang, S., Spatial and Temporal Variations of Gaseous and Particulate Pollutants in Six Sites in Tibet, China, during 2016–2017. *Aerosol and Air Quality Research* **2019,** *19*, (3), 516-527.
4. Wu, S. S.; Huang, B.; Wang, J. H.; He, L. J.; Wang, Z. Y.; Yan, Z.; Lao, X. Q.; Zhang, F.; Liu, R. Y.; Du, Z. H., Spatiotemporal mapping and assessment of daily ground NO2 concentrations in China using high-resolution TROPOMI retrievals. *Environ Pollut* **2021,** *273*, 116456.
5. Wang, Y.; Yuan, Q. Q.; Li, T. W.; Zhu, L. Y.; Zhang, L. P., Estimating daily full-coverage near surface O-3, CO, and NO2 concentrations at a high spatial resolution over China based on S5P-TROPOMI and GEOS-FP. *Isprs J Photogramm* **2021,** *175*, 311-325.
6. Li, L. F.; Wu, J. J., Spatiotemporal estimation of satellite-borne and ground-level NO2 using full residual deep networks. *Remote Sens Environ* **2021,** *254*, 112257.
7. Jiang, Q. T.; Christakos, G., Space-time mapping of ground-level PM2.5 and NO2 concentrations in heavily polluted northern China during winter using the Bayesian maximum entropy technique with satellite data. *Air Qual Atmos Hlth* **2018,** *11*, (1), 23-33.
8. Zhan, Y.; Luo, Y. Z.; Deng, X. F.; Zhang, K. S.; Zhang, M. H.; Grieneisen, M. L.; Di, B. F., Satellite-Based Estimates of Daily NO2 Exposure in China Using Hybrid Random Forest and Spatiotemporal Kriging Model. *Environ Sci Technol* **2018,** *52*, (7), 4180-4189.
9. Qin, K.; Han, X.; Li, D. H.; Xu, J.; Loyola, D.; Xue, Y.; Zhou, X. R.; Li, D.; Zhang, K. F.; Yuan, L. M., Satellite-based estimation of surface NO2 concentrations over east-central China: A comparison of POMINO and OMNO2d data. *Atmos Environ* **2020,** *224*, 117322.
10. Dou, X.; Liao, C.; Wang, H.; Huang, Y.; Tu, Y.; Huang, X.; Peng, Y.; Zhu, B.; Tan, J.; Deng, Z.; Wu, N.; Sun, T.; Ke, P.; Liu, Z., Estimates of daily ground-level NO2 concentrations in China based on Random Forest model integrated K-means. *Advances in Applied Energy* **2021,** *2*, 100017.
11. Chi, Y. L.; Fan, M.; Zhao, C. A. F.; Yang, Y. K.; Fan, H.; Yang, X. C. A.; Yang, J.; Tao, J. H., Machine learning-based estimation of ground-level NO2 concentrations over China. *Sci Total Environ* **2022,** *807*, 150721.
12. Xu, H.; Bechle, M. J.; Wang, M.; Szpiro, A. A.; Vedal, S.; Bai, Y. Q.; Marshall, J. D., National PM2.5 and NO2 exposure models for China based on land use regression, satellite measurements, and universal kriging. *Sci Total Environ* **2019,** *655*, 423-433.
13. Chen, Z. Y.; Zhang, R.; Zhang, T. H.; Ou, C. Q.; Guo, Y. M., A kriging-calibrated machine learning method for estimating daily ground-level NO2 in mainland China. *Sci Total Environ* **2019,** *690*, 556-564.
14. Qin, K.; Rao, L. L.; Xu, J.; Bai, Y.; Zou, J. H.; Hao, N.; Li, S. S.; Yu, C., Estimating Ground Level NO2 Concentrations over Central-Eastern China Using a Satellite-Based Geographically and Temporally Weighted Regression Model. *Remote Sens-Basel* **2017,** *9*, (9), 950.
15. Liu, J. J., Mapping high resolution national daily NO2 exposure across mainland China using an ensemble algorithm. *Environ Pollut* **2021,** *279*, 116932.
